# Bacterial reporter–paired scRNA sequencing reveals cross talk between zinc starvation and zinc toxicity in macrophage antibacterial defense

**DOI:** 10.1073/pnas.2530503123

**Published:** 2026-03-09

**Authors:** Jessica B. von Pein, Stacey B. Andersen, Jon Xu, Minh-Duy Phan, Emma K. Dalton, Michael Koczerka, Claudia J. Stocks, James E. B. Curson, Zoe Vandeleur, Nicholas D. Condon, Steven J. Hancock, Christian M. Nefzger, Nathan J. Palpant, Divya Ramnath, Ronan Kapetanovic, Mark A. Schembri, Matthew J. Sweet

**Affiliations:** ^a^Institute for Molecular Bioscience, The University of Queensland, Brisbane, QLD 4072, Australia; ^b^Institute for Molecular Bioscience Centre for Cell Biology of Chronic Disease, The University of Queensland, Brisbane, QLD 4072, Australia; ^c^Genome Innovation Hub, The University of Queensland, Brisbane, QLD 4072, Australia; ^d^The University of Queensland Sequencing Facility, The University of Queensland, Brisbane, QLD 4072, Australia; ^e^School of Chemistry and Molecular Biosciences, The University of Queensland, Brisbane, QLD 4072, Australia; ^f^Australian Infectious Diseases Research Centre, The University of Queensland, Brisbane, QLD 4072, Australia; ^g^Infectiologie et Santé Publique, Institut national de recherche pour l’agriculture, l’alimentation et l’environnement, Université de Tours, Nouzilly 37380, France

**Keywords:** single-cell RNA sequencing, macrophage, *E. coli*, zinc toxicity, *SLC30A4/ZNT4*

## Abstract

Mechanisms by which macrophages deploy antibacterial zinc toxicity are poorly understood. To gain insight into this antimicrobial pathway, we developed bacterial reporter–paired single-cell RNA sequencing of human monocyte-derived macrophages (HMDM) infected with an *Escherichia coli* zinc-stress reporter strain. We identified HMDM subpopulations harboring zinc-stressed *E. coli* and corresponding mammalian genes predicted to be associated with either zinc toxicity or survival of zinc-stressed bacteria. Consistent with the latter, *SLC30A4* that encodes zinc exporter ZNT4 was enriched in one subpopulation of HMDM containing zinc-stressed *E. coli* and its overexpression in human macrophages increased intracellular *E. coli* survival. At a population level, *SLC30A4* expression was rapidly downregulated in human macrophages responding to *E. coli* and its ectopic expression in macrophages attenuated zinc starvation of intracellular *E. coli*. This is consistent with a model in which macrophages switch off SLC30A4 to engage zinc starvation, while also deploying zinc toxicity against bacteria adapting to a low-zinc environment. Consistent with this, intramacrophage *E. coli* rapidly upregulated *znuA* messenger RNA (mRNA) that is induced during zinc limitation, with *zntA* mRNA that is induced during zinc stress peaking later. Moreover, *E. coli* cultured under conditions of zinc limitation displayed greatly enhanced zinc sensitivity. Susceptibility of zinc-sensitive *E. coli* to killing by macrophages was also attenuated when zinc uptake by *E. coli* was inactivated, confirming the coordinated actions of zinc starvation and zinc toxicity in macrophage antibacterial responses. Strategies that enhance zinc starvation of intracellular bacteria could be exploited in the design of host-directed therapeutics that amplify macrophage-mediated antibacterial zinc toxicity.

## Significance

We developed high-throughput bacterial reporter–paired single-cell RNA sequencing (scRNA-seq) to track both mammalian and bacterial gene expression in single cells and identified macrophage gene signatures associated with zinc toxicity, as well as those associated with survival of intracellular zinc-stressed bacteria. Through this approach, we found that macrophages starve intracellular *Escherichia coli* of zinc to sensitize them to subsequent zinc toxicity. Zinc starvation and zinc toxicity have not previously been linked within a single antimicrobial response, so these findings represent a major advance in our understanding of innate immune antibacterial defense. The workflow developed here can be adapted to track pathogen responses to the intracellular environment, providing insights into host–pathogen interactions and opportunities for devising host-directed therapies to combat bacterial infections.

Macrophages use phagocytosis to engulf and destroy pathogens, as well as pattern recognition receptors such as the toll-like receptors (TLRs) to engage downstream signaling. Signaling via TLRs induces antimicrobial effector genes, including those that regulate metal ion availability, to assist in pathogen clearance (1, 2). Metal ion availability contributes to antimicrobial defense either through sequestration away from pathogens to limit their growth and survival (3) or through delivery of toxic concentrations for direct microbial poisoning (4).

Zinc has essential roles in many processes, including immune responses (5). Innate immune cells can mobilize zinc during infection to sequester this metal ion away from pathogens (6) or to direct toxic concentrations of zinc toward pathogens (7). Human macrophages deploy zinc toxicity against pathogens including *Mycobacterium tuberculosis* (8), *Salmonella enterica* (9), and *Escherichia coli* (10), but mechanisms by which this pathway is engaged are incompletely understood. TLR signaling mobilizes zinc and promotes zinc vesicle formation in innate immune cells (9, 11), suggesting that pathogen detection systems trigger gene expression changes to engage zinc toxicity.

Macrophage zinc homeostasis is controlled by the zinc-importing solute carrier (SLC) 39A family of transporters, SLC30A/ZNT proteins that export zinc, and metal-binding proteins such as metallothioneins (MTs) that buffer cytosolic zinc. TLR agonists and/or bacterial challenge induce the expression of zinc transporters and MTs in innate immune cells (8, 12, 13), consistent with a role for regulated gene expression in zinc toxicity. SLC30A zinc exporters are likely facilitators of zinc delivery into intracellular compartments containing internalized pathogens (7). Indeed, ectopic SLC30A1/ZNT1 expression in macrophage-like THP-1 cells drives zinc vesicle formation, enhances zinc stress in intracellular *E. coli*, and promotes bacterial killing by macrophages (13). A role for myeloid SLC30A1/ZNT1 in antibacterial defense has also been confirmed in a *Salmonella* challenge model in mice (14). The cystic fibrosis transmembrane regulator (CFTR) is also required for macrophage-mediated antibacterial zinc toxicity (15), but other host factors regulating the zinc toxicity antimicrobial pathway have not yet been described.

Here, we developed high-throughput bacterial reporter–paired single-cell RNA sequencing (HTBRP scRNA-seq) of both macrophages and intracellular *E. coli* to investigate mechanisms of antibacterial zinc toxicity. We identify macrophage subpopulations containing zinc-stressed *E. coli* and candidate genes that either facilitate zinc toxicity or permit intramacrophage survival of zinc-stressed bacteria. Through studies on one such gene, we reveal functional cooperativity between zinc starvation and zinc toxicity in macrophage antibacterial defense.

## Results

### Heterogeneity of the Zinc Toxicity Response in HMDM.

A previous study on dual reporter *E. coli* that constitutively express GFP and zinc-inducible (*zntA*-dependent) mCherry revealed that intracellular *E. coli* experience zinc stress within HMDM (10). This study also showed that a nonpathogenic *E. coli* K-12 strain is susceptible to macrophage zinc toxicity, whereas a pathogenic *E. coli* strain evades this response. We sought to exploit this system in single-cell transcriptomics for identification of macrophage genes regulating the zinc toxicity response, using the *E. coli* K-12 strain to ensure that pathogen evasion strategies did not interfere with our screen. We began by reducing background mCherry expression that may confound data interpretation in transcriptomic studies. The *zntA* promoter is repressed by apo-zntR under zinc-limiting conditions (16) but enhanced by zinc-bound zntR when zinc concentrations are high (17). Compared to the original dual reporter *E. coli* strain, incorporation of a *zntR* cassette reduced basal mCherry expression in bacteria alone (*SI Appendix*, Fig. S1 *A* and *B*) with inducible mCherry expression in infected HMDM being retained (*SI Appendix*, Fig. S1*C*), desirable features for transcriptomic studies tracking zinc toxicity.

**Fig. 1.**
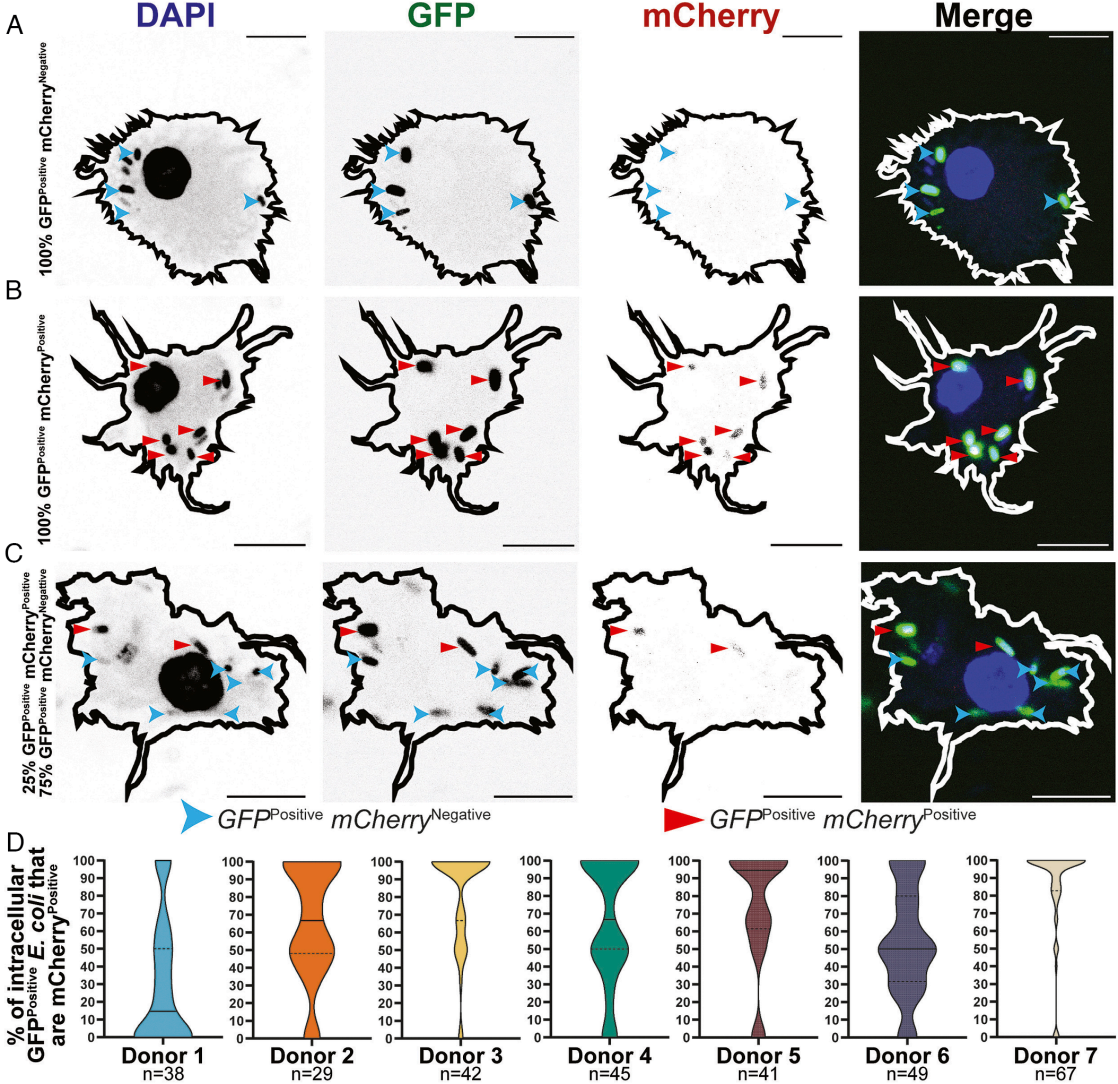
HMDM exhibit heterogenous zinc toxicity profiles. HMDM were infected with zntR-dual reporter *E. coli* (MOI 100) for 8 h and imaged via confocal microscopy. (*A*–*C*) Images are from a single representative experiment from seven independent donors (n = 7). (Scale bar, 10 µm.) mCherry^Positive^ and mCherry^Negative^ bacteria are indicated with straight red and curved blue arrowheads, respectively. Macrophage outlines were mapped based on background DAPI stain. (*D*) The % of GFP^Positive^ bacteria that are mCherry^Positive^ per HMDM obtained by seven human donors, with median and quartiles represented by solid and dotted lines, respectively. Numbers (n) of HMDMs assessed for each donor are indicated.

Confocal microscopy of the zntR-dual reporter *E. coli* in HMDM revealed heterogeneity in the zinc toxicity response. Macrophages were identified with all intracellular GFP^Positive^ *E. coli* being mCherry^Negative^ (no zinc stress, Fig. 1*A*), all intracellular GFP^Positive^ *E. coli* being mCherry^Positive^ (zinc stress, Fig. 1*B*) or intracellular GFP^Positive^ *E. coli* being a mixture of mCherry^Negative^ and mCherry^Positive^ (mixed zinc stress, Fig. 1*C*). Quantification of intracellular GFP^Positive^ mCherry^Positive^ zntR-dual reporter *E. coli* across individual HMDM donors revealed that the zinc toxicity response was heterogenous across all donors examined (Fig. 1*D*). We sought to exploit this heterogeneity through HTBRP scRNA-seq to reveal mechanistic insight.

### HTBRP scRNA-seq to Track Zinc Toxicity in HMDM.

To assess antibacterial zinc toxicity in HMDM at a single-cell level, HTBRP scRNA-seq was developed. The 10× Genomics Chromium platform was utilized, taking advantage of its high sensitivity and unbiased, high-throughput methodology (18). In our workflow, the presence of *GFP* transcripts indicates intracellular *E. coli* within macrophages, while detection of both *GFP* and *mCherry* transcripts indicates macrophages containing zinc-stressed *E. coli* (*SI Appendix*, Fig. S2*A*). Feature barcode sequences were inserted into the C-terminal region of the *GFP* and *mCherry* genes in the zntR-dual reporter construct (*SI Appendix*, Fig. S2*B*) to allow for transcript enrichment during the 10× Genomics workflow. HMDM were spin-infected with barcoded zntR-dual reporter *E. coli* for 2 and 6 h (*SI Appendix*, Fig. S2*C*) and a modified 10× Genomics Chromium workflow was used to attain libraries for sequencing (*SI Appendix*, Fig. S2 *D*–*G*). Metrics for scRNA-seq are presented in Dataset S1.

To validate our approach (*SI Appendix*, Fig. S2 *B*–*G*), multiple quality control measures were employed. Barcoded zntR-dual reporter *E. coli* were zinc-responsive in culture, as determined by flow cytometry (*SI Appendix*, Fig. S3*A*). Spin-infection conditions for HTBRP scRNA-seq did not affect macrophage viability (*SI Appendix*, Fig. S3*B*). Lifted HMDM populations were viable (*SI Appendix*, Fig. S3*C*) and approximately 70% and 35% of HMDM lifted after 2 h and 6 h postinfection (p.i.) contained detectable bacterial GFP signal, respectively (*SI Appendix*, Fig. S3*D*). In addition, we confirmed that intramacrophage *E. coli* were viable at the timepoints used for scRNA-seq (*SI Appendix*, Fig. S3*E*, cells from the same donor as used in scRNA-seq were infected in parallel). HMDM transcriptomes were labeled according to human embryonic hematopoietic cell marker genes, as previously defined (19). This classification system is based on transcriptome profiles defined from human embryonic tissues (19) and does not reflect overall HMDM purity. Nonetheless, >50% of the HMDM population sampled were identified as “macrophage-like,” with the remainder of the infected HMDM population identified as either “monocyte-” or “other macrophage progenitor-like” (*SI Appendix*, Fig. S3 *F* and *G*). These HMDM populations were therefore used for subsequent HTBRP scRNA-seq analyses.

### The Zinc Toxicity Response Occurs in Subpopulations of *E. coli*–Infected HMDM.

**Fig. 2.**
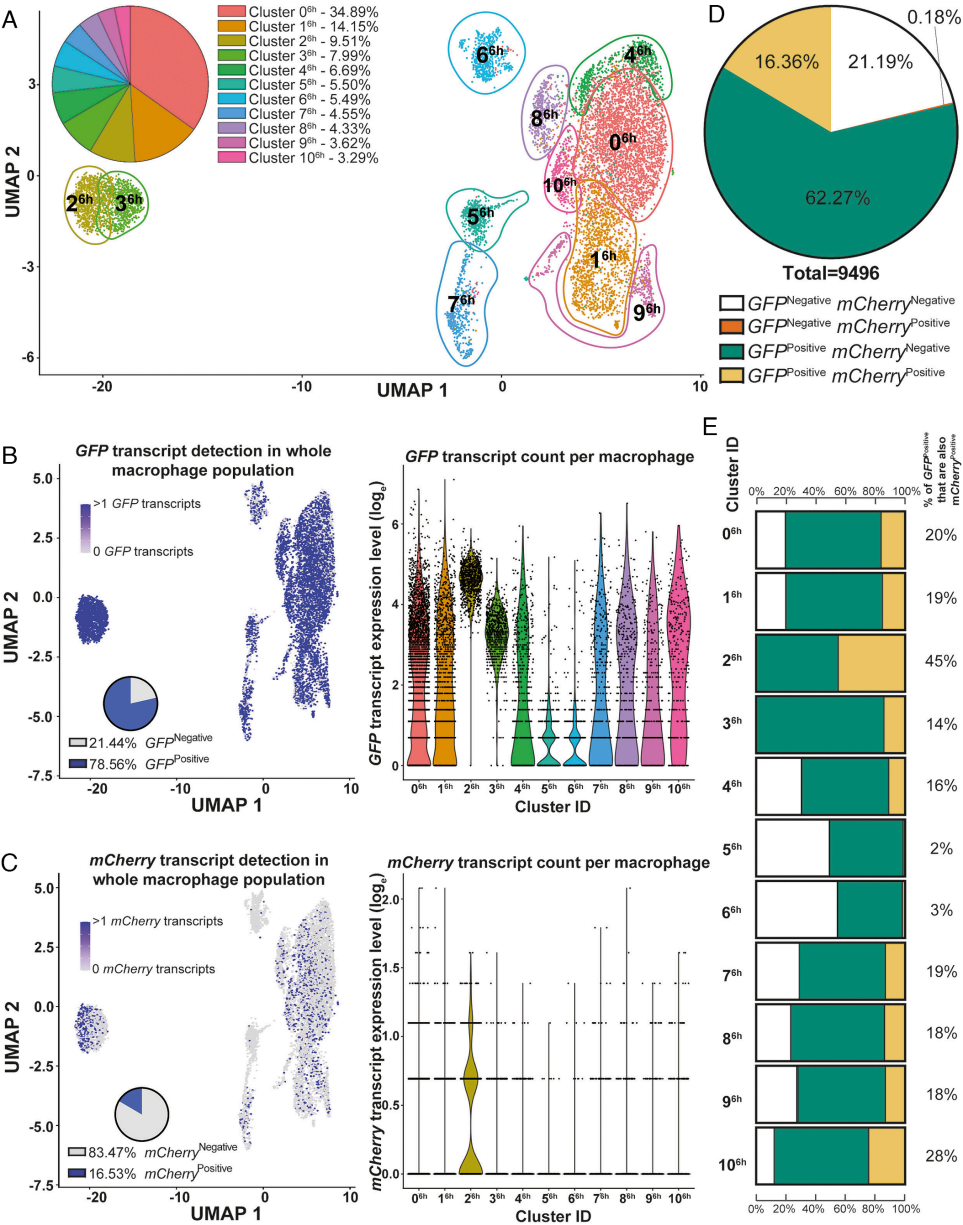
HTBRP scRNA-seq reveals HMDM subpopulations engage zinc toxicity at 6 h postinfection. HTBRP scRNA-seq and analysis of HMDMs infected with barcoded zntR-dual reporter *E. coli* (MOI 30) for 6 h. (*A*) Proportions of overall HMDM population for each gene expression UMAP cluster (n = 9,496 HMDMs). *GFP* (*B*, *Left*) and *mCherry* (*C*, *Left*) transcript distributions were overlaid onto mammalian gene expression data with % of *GFP*^Positive^/*mCherry*^Positive^ HMDM in the total population shown within *Insets*. Log-transformed *GFP* (*B*, *Right*) and *mCherry* (*C*, *Right*) transcript counts from individual HMDM, grouped according to UMAP clusters. (*D* and *E*) HMDM *GFP* and *mCherry* expression profiles are represented as percentages of macrophages in the total population (*D*), and within UMAP clusters in bar schematics (*E*). The percentage of *GFP*^Positive^ HMDMs in each cluster that were *mCherry*^Positive^ is indicated on the *Right* (*E*).

At 2 h p.i., <3% of HMDM transcriptome profiles in clusters 7^2h^ and 10^2h^ were distinct from other HMDM subpopulations (*SI Appendix*, Fig. S4*A*). Conversely, at 6 h p.i., HMDM subpopulations that were highly (2^6h^ and 3^6h^) and moderately (5^6h^, 6^6h^, and 7^6h^) distinct from centralized clusters constituted ~17.5% and ~15.5% of the HMDM sampled, respectively (Fig. 2*A*). *GFP* transcripts were identified in ~66% (*SI Appendix*, Fig. S4*B*) and ~78% of HMDM (Fig. 2*B*) at 2 h and 6 h p.i., respectively, indicating high rates of detection for this constitutive bacterial reporter gene. The sensitivity of *GFP* mRNA detection via scRNA-seq at 6 h p.i. (~78%, Fig. 2*B*) was higher than that detected via flow cytometry (~35%, *SI Appendix*, Fig. S3*D*). This increased sensitivity is not surprising, given that our scRNA-seq method directly measures bacterial reporter mRNA transcripts, whereas flow cytometry measures overall HMDM fluorescence intensity, sourced from intracellular bacteria within complex intramacrophage compartments and undergoing antimicrobial attack (*SI Appendix*, Fig. S3*E*). Next, *mCherry* transcripts were used to identify zinc-stressed bacteria within 2 h-infected HMDM. This analysis revealed that only ~7.7% of HMDM contained identifiable *mCherry* transcripts at 2 h p.i. (*SI Appendix*, Fig. S4 *C*–*E*). Cluster 0^2h^ contained the highest proportion (14%) of *GFP*^Positive^ HMDM that were also *mCherry*^Positive^ in a single cluster (*SI Appendix*, Fig. S4*E*). This suggests low engagement of the zinc toxicity response at early stages of infection. By 6 h p.i., ~16.5% of HMDM had detectable *mCherry* transcripts (Fig. 2 *C* and *D*), with the highest proportions of *GFP*^Positive^ HMDM that were also *mCherry*^Positive^ in single clusters being 45% (cluster 2^6h^) and 28% (cluster 10^6h^, Fig. 2*E*). This is consistent with engagement of the zinc toxicity response at later stages of infection. We note that, at 6 h p.i., 21.19% HMDM were both *GFP*^Negative^ and *mCherry*^Negative^ (Fig. 2*D*). This could reflect cells that were refractory to infection and/or highly efficient at killing ingested *E. coli*. It is also possible that some *E. coli* lost the reporter plasmid, so 21.19% of cells may be an overrepresentation of the true percentage of uninfected cells.

We next focused on cluster 2^6h^ that contained the highest proportion of *GFP*^Positive^ and *mCherry*^Positive^ HMDM in the 6 h p.i. dataset (Fig. 2*E*). *GFP* and *mCherry* transcript counts did not correlate in any HMDM cluster (*SI Appendix*, Fig. S5*A*), indicating that *mCherry* transcript enrichment in cluster 2^6h^ could not simply be explained by high intracellular *E. coli* loads in these HMDM (Fig. 2 *B*, *Right*). Furthermore, although cluster 2^6h^ and 3^6h^ closely integrated in Uniform Manifold Approximation and Projection (UMAP) clustering (Fig. 2*A*), cluster 3^6h^ had high levels of *GFP* transcripts (Fig. 2 *B*, *Right*) but much lower levels of *mCherry* transcripts (Fig. 2 *C*, *Right*, Fig. 2*E*). Together, these data identify cluster 2^6h^ as a distinct HMDM population with bacterial signatures indicative of high levels of zinc stress during infection.

### Macrophage Transcriptomes Associated with the Macrophage Zinc Toxicity Response.

**Fig. 3.**
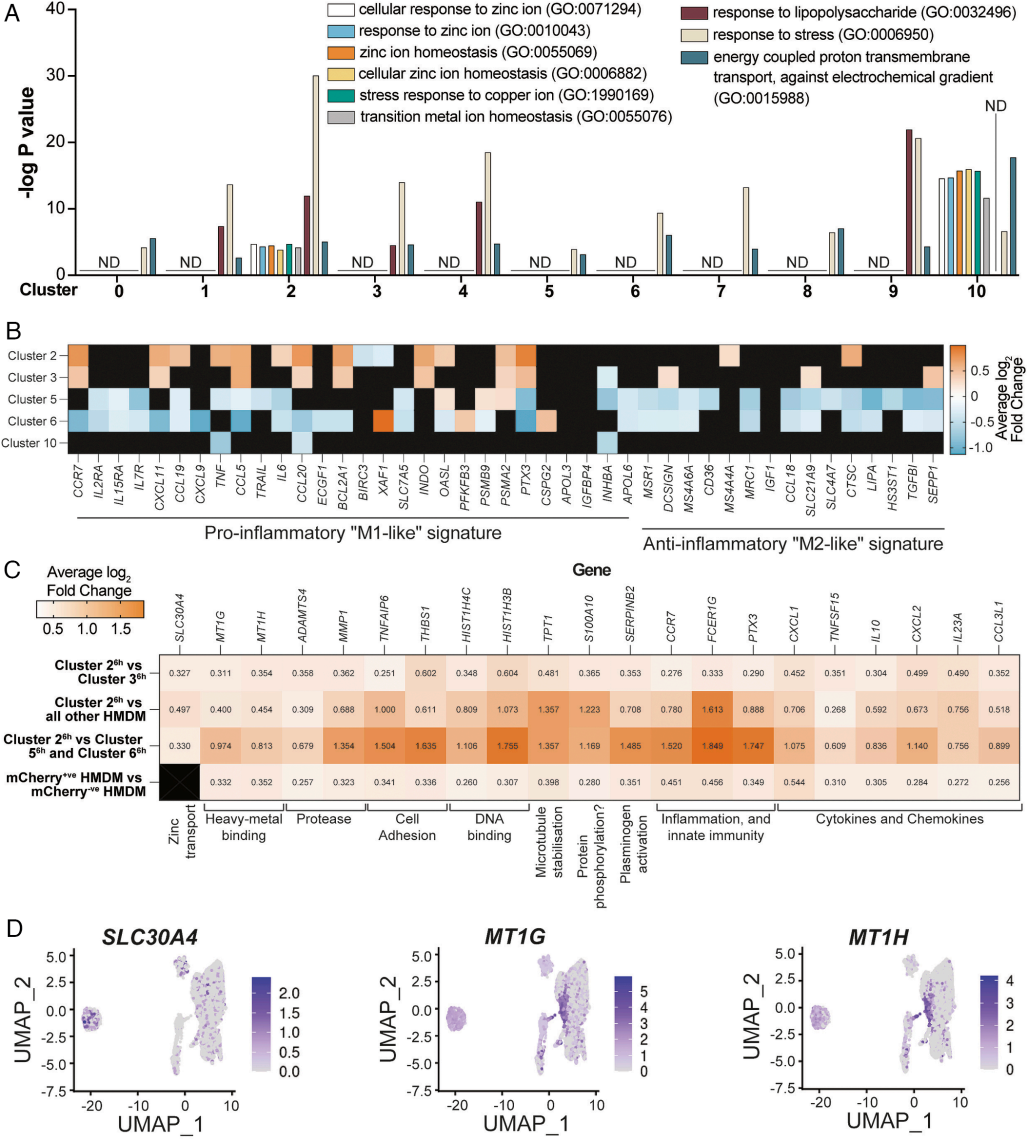
Zinc homeostasis pathways and zinc-related genes associate with zinc stress signatures. (*A*) −log *P* values from GO analysis pathways identified from DEGs per HMDM cluster defined in Fig. 2*A*. “ND” indicates pathways not detected in GO analysis of a cluster’s DEGs. (*B*) Heatmap of DEG profiles within infected HMDM clusters, according to proinflammatory (“M1-like”) and anti-inflammatory (“M2-like”) signatures that have previously been defined (20). (*C*) Heatmap of the average log_2_ fold change of expression of DEGs in specified HMDM clusters. Numbers in individual boxes indicate the average log_2_ fold change in expression of individual genes for the specific comparisons in each row. (*B* and *C*) Genes not detected as a DEG in clusters/comparisons are represented with a black square. (*D*) Expression overlay for candidate genes specified in (*C*).

Dataset S2 contains the complete list of differentially expressed genes (DEGs) identified for HMDM clusters at 6 h p.i., as defined in Fig. 2*A*. Gene ontology (GO) analysis was used to determine if zinc-related pathways were enriched in DEGs for HMDM subpopulations containing zinc-stressed bacteria. Whereas generalized pathways such as “response to stress” were upregulated in all HMDM clusters at 6 h p.i. (Fig. 3*A*), several pathways relating to metal ions, including zinc and copper, were enriched in only clusters 2^6h^ and 10^6h^ (Fig. 3*A*) that were also enriched for zinc-stressed *E. coli* (Fig. 2*E*). Of note, *SLC30A1* was enriched in cluster 10^6h^ (Dataset S2), consistent with our previous observations that this zinc exporter promotes antibacterial zinc toxicity in macrophages (13). Collectively, these findings validate the HTBRP scRNA-seq approach developed here.

As cluster 2^6h^ had the most striking bacterial zinc stress profile (Fig. 2*E*), we next assessed DEGs identified in this cluster. Initial comparisons were made against cluster 3^6h^ that closely integrated with cluster 2^6h^ in UMAP clustering (Fig. 2*A*) but displayed a distinct bacterial zinc stress profile (Fig. 2*E*). GO pathway analysis of DEGs for clusters 2^6h^ and 3^6h^ revealed shared enrichment in specific genetic pathways (e.g., antigen processing and presentation, reactive oxygen species metabolism) and depletion of others (e.g., leukocyte apoptosis, cell cycle progression) (Dataset S3). Both clusters 2^6h^ and 3^6h^ had gene signatures associated with macrophage inflammatory responses, although the magnitude of upregulation and the number of “proinflammatory” DEGs [as previously defined (20)] were increased in cluster 2^6h^ when compared with cluster 3^6h^ (Fig. 3*B*). Interestingly, cluster 10^6h^ that was also enriched for zinc-stressed bacteria did not have an inflammatory gene signature, while inflammatory genes were generally downregulated in clusters 5^6h^ and 6^6h^ that contained the lowest proportion of zinc-stressed bacteria (Fig. 3*B*). This was also the case for several other HMDM clusters, suggesting that the proinflammatory profiles of clusters 2^6h^ and cluster 3^6h^ are distinct from the other HMDM populations (*SI Appendix*, Fig. S5*B*). One potential explanation for the proximity of clusters 2^6h^ and 3^6h^ (Fig. 2*A*) is that HMDM in cluster 3^6h^ could represent a dynamic macrophage state engaged either immediately prior to, or after, the zinc toxicity response observed in cluster 2^6h^. However, RNA velocities (21) for HMDM in cluster 3^6h^ and 2^6h^ did not integrate (*SI Appendix*, Fig. S5*C*), suggesting these clusters do not represent macrophage transition states. Nonetheless, they may represent macrophage populations engaging distinct antimicrobial responses, one being zinc toxicity, in response to infection. We therefore next sought to understand whether HMDM within cluster 2^6h^ effectively engage zinc toxicity.

To identify genes associated with the macrophage zinc toxicity response, DEGs from HMDM populations enriched for zinc stress signatures were compared with other HMDM populations. These comparisons included i) HMDM in cluster 2^6h^ compared to HMDM in cluster 3^6h^; ii) HMDM in cluster 2^6h^ compared to all other HMDM at 6 h p.i.; iii) HMDM in cluster 2^6h^ compared to HMDM in clusters 5^6h^ and 6^6h^ that were less “proinflammatory” than clusters 2^6h^ and 3^6h^ (Fig. 3*B*) and that had very low *mCherry* and *GFP* expression (Fig. 2 *B*, *C*, and *E*); and iv) *mCherry*^Positive^ HMDM compared with *mCherry*^Negative^ HMDM in the whole dataset, independent of HMDM subpopulation (Fig. 3*C*). Dataset S4 shows all DEGs detected in at least two of the comparisons. Twenty DEGs, including metallothionein-encoding *MT1G* and *MT1H*, were upregulated in all four comparisons (Fig. 3*C*) and 141 DEGs were upregulated in three of four comparisons (Dataset S4). The latter includes the zinc exporter *SLC30A4*/*ZNT4* that was enriched in Cluster 2^6h^ [45% of HMDM in this cluster had a detectable bacterial zinc stress signature (Fig. 2*E*)] and that has known roles in zinc trafficking and host defense in macrophage antimicrobial responses (22). Thus, the zinc-associated genes *SLC30A4*, *MT1G,* and *MT1H* are expressed by cluster 2^6h^ macrophages that have engaged the zinc toxicity response. Expression overlay plots confirmed that *SLC30A4*, *MT1G,* and *MT1H* expression was most prominent in clusters 10^6h^ and/or 2^6h^ (Fig. 3*D*), aligning with our observation that these clusters were associated with metal ion pathways (Fig. 3*A*). *SLC30A4* was the only metal transporter identified in DEG comparisons for cluster 2^6h^ (Fig. 3*C* and Dataset S4) and its expression was largely restricted to cluster 2^6h^ (Fig. 3*D*). *SLC30A4* was thus selected for further investigation into its role in macrophage-mediated zinc toxicity.

### Ectopic SLC30A4 Expression Impairs Zinc Starvation and *E. coli* Killing by Macrophages.

**Fig. 4.**
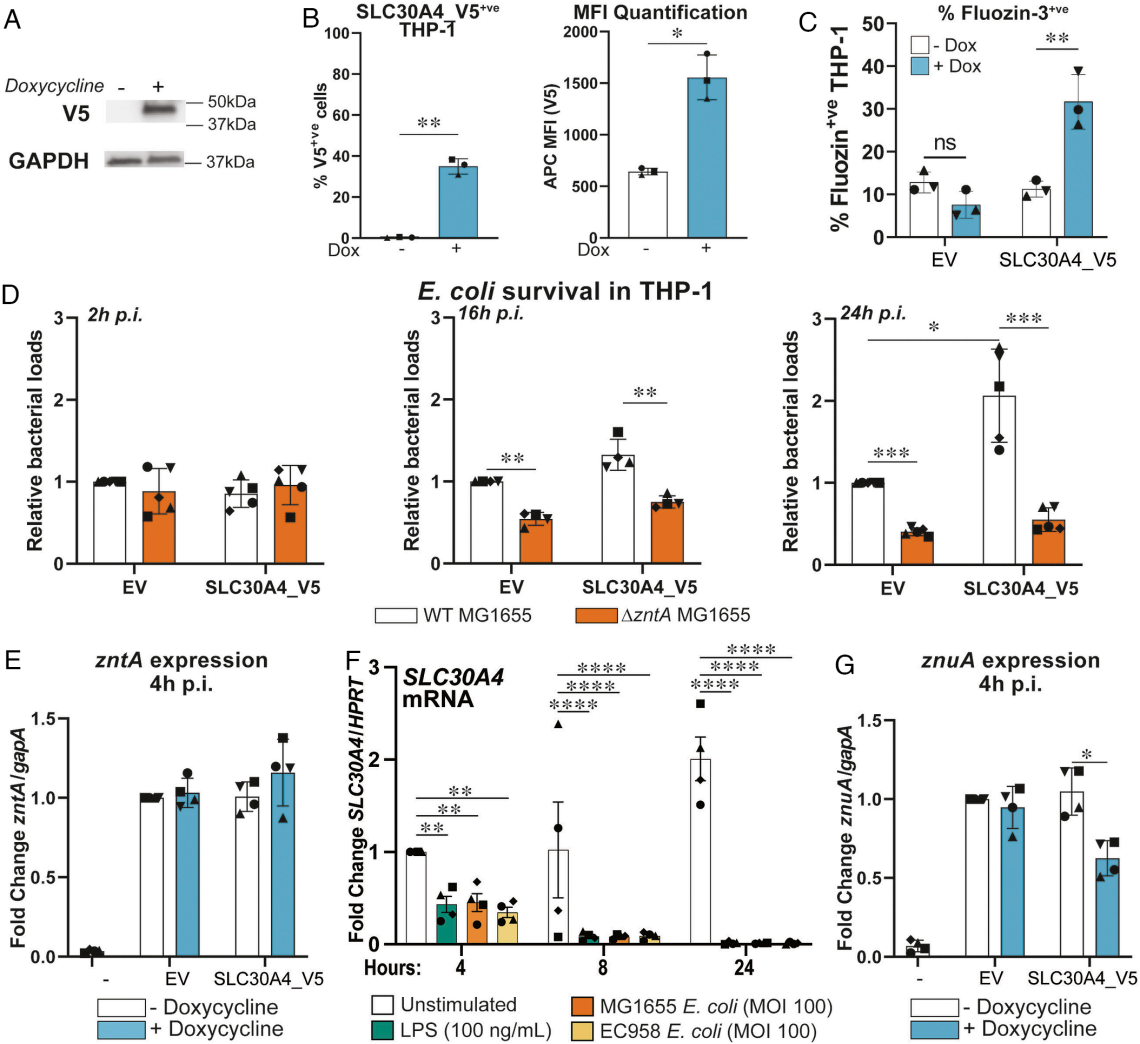
Ectopic SLC30A4 expression inhibits macrophage antimicrobial activity and zinc starvation. Lentiviral EV or SLC30A4_V5 differentiated THP-1 cells were stimulated with 100 ng/mL Dox for 16 h. (*A*) Immunoblot of THP-1 lysate, representative of four experiments (n = 4). (*B* and *C*) THP-1 cells were fixed and stained for V5 (*B*) or Fluozin-3 (*C*) and assessed via flow cytometry. The % V5^Positive^ (V5^+ve^) THP-1 and median fluorescent intensity (MFI) of all cells (*B*) and % Fluozin-3^+’ve^ THP-1 cells (*C*) are presented as mean ± SD from three (n = 3) independent experiments. (*D*) Dox-stimulated THP-1 were infected with wild-type (WT) or Δ*zntA E. coli* and intracellular bacterial loads (CFU/mL) were assessed at indicated time points. Bacterial loads are presented relative to EV THP-1. (*E*) Differentiated THP-1 ± Dox were infected with WT *E. coli* for 4 h, then *zntA* mRNA levels were quantified by qPCR. Control samples (−) represent bacteria cultured in THP-1 media for 2 h. Fold change expression was calculated relative to unstimulated EV THP-1 cells. (*F*) HMDMs were stimulated with LPS or infected with *E. coli* (nonpathogenic MG1655 or uropathogenic EC958) and *SLC30A4* mRNA levels were quantified by qPCR, presented as fold change relative to unstimulated HMDM at 4 h. (*G*) Similar to (*E*), differentiated THP-1 ± Dox were infected with WT *E. coli* for 4 h, then *znuA* mRNA levels were quantified by qPCR. (*D*–*G*) Data represent mean ± SD of four or five (n = 4-5) independent experiments, indicated by different symbols. Statistical tests were performed on raw (non-normalized) data using the two-tailed *t* test (*B*), two-way ANOVA with Benjamini–Hochberg false discovery rate correction (*C* and *D*), Sidak (*E* and *G*), or Tukey (*F*) multiple comparisons tests. Adjusted *P* values are indicated as *P < 0.05, **P < 0.01, ***P < 0.001 and ****P < 0.0001, all other comparisons were not significant.

Since only subpopulations of HMDM expressed SLC30A4 (Fig. 3*D*) and were associated with zinc toxicity (Fig. 2*C*), we considered that gene silencing approaches would be unlikely to reveal clear phenotypes at a whole population level. We therefore adopted a doxycycline (Dox)-inducible system for overexpressing C-terminal V5-tagged SLC30A4 to assess macrophage zinc toxicity responses against *E. coli*. Dox-induced SLC30A4 overexpression in PMA-differentiated THP-1 cells (Fig. 4*A*) resulted in ~40% of cells expressing SLC30A4-V5 (Fig. 4 *B*, *Left*) and increased available intracellular zinc as assessed by Fluozin-3 staining (Fig. 4*C*). Similarly, mRNA-mediated ectopic expression of SLC30A4 in THP-1 cells (*SI Appendix*, Fig. S6*A*) led to increased Fluozin-3 staining by comparison to vehicle-transfected control cells (*SI Appendix*, Fig. S6*B*). The Fluozin-3 staining pattern (*SI Appendix*, Fig. S6*B*) was similar to that observed for ectopically expressed SLC30A4 (anti-V5, *SI Appendix*, Fig. S6*C*) and likely reflects Golgi staining. This would be consistent with the known role of SLC30A4 in shuttling zinc into the Golgi (23) and/or phagosomes (22), rather than increasing zinc uptake into cells. Indeed, inductively coupled plasma mass spectrometry (ICP-MS) revealed that overexpression of SLC30A4 did not increase total intracellular zinc levels in THP-1 cells (*SI Appendix*, Fig. S6*D*), although total magnesium levels were reduced and there were also trends for reduced levels of other metal ions (*SI Appendix*, Fig. S6*E*). Collectively, these findings suggest that SLC30A4 expression in macrophages increases pools of available zinc, aligning with the enrichment of *SLC30A4* mRNA expression in a HMDM subpopulation containing zinc-stressed bacteria (Fig. 3*C*). Next, the effect of Dox-induced SLC30A4 expression on killing of a zinc-sensitive (Δ*zntA*) *E. coli* mutant in PMA-differentiated THP-1 cells was assessed. Consistent with our previous study (13), intracellular survival of the Δ*zntA* mutant was significantly reduced at 16 h and 24 h p.i. in empty vector (EV) control THP1 cells (Fig. 4*D*). However, overexpressing SLC30A4_V5 did not reduce *E. coli* loads in these cells (Fig. 4*D* and *SI Appendix*, Fig. S7*A*). This suggests that SLC30A4 does not promote zinc-mediated bacterial clearance, even though this zinc transporter did increase available zinc levels in macrophages (Fig. 4*C* and *SI Appendix*, Fig. S6*B*). In line with this, intramacrophage *E. coli* did not display enhanced zinc stress when SLC30A4_V5 was overexpressed in THP-1 cells (Fig. 4*E*). Instead, SLC30A4 overexpression significantly increased intracellular *E. coli* loads at 24 h p.i. (Fig. 4*D* and *SI Appendix*, Fig. S7*A*). Thus, ectopic SLC30A4 expression enables intracellular survival of zinc-stressed *E. coli*, suggesting a surprising model that some genes expressed by macrophages harboring zinc-stressed bacteria may actually support bacterial survival. Consistent with this, *SLC30A4* mRNA was markedly downregulated in HMDM responding to LPS or *E. coli* (Fig. 4*F* and *SI Appendix*, Fig. S7*B*). We postulate that this downregulation of *SLC30A4* during bacterial infection may permit an optimal macrophage antibacterial response.

We next considered that downregulation of *SLC30A4* after *E. coli* challenge may be linked to a zinc sequestration antimicrobial response. The ZnuA zinc importer is upregulated in *E. coli* when zinc availability is limited (24). Here, we found that switching on SLC30A4_V5 in THP-1 cells reduced zinc starvation against intracellular *E. coli*, as assessed by *znuA* mRNA levels (Fig. 4*G*). This suggests that ectopic SLC30A4 expression antagonizes the initiation of a zinc starvation response against intracellular *E. coli*, hence facilitating their survival within macrophages. Thus, the early downregulation of *SLC30A4* in macrophages (Fig. 4*F*) likely enables macrophages to engage zinc starvation of intracellular *E. coli*. This starvation step could potentially sensitize intracellular *E. coli* to the subsequent zinc toxicity response, thus supporting bacterial killing by macrophages. This could explain why zinc-stressed *E. coli* were able to survive in HMDM subpopulations with upregulated *SLC30A4* (Fig. 3*C*) and suggests that SLC30A4 may function as a molecular switch coordinating zinc starvation and zinc toxicity.

### The Macrophage Zinc Toxicity Response against Intracellular *E. coli* Requires Zinc Starvation.

**Fig. 5.**
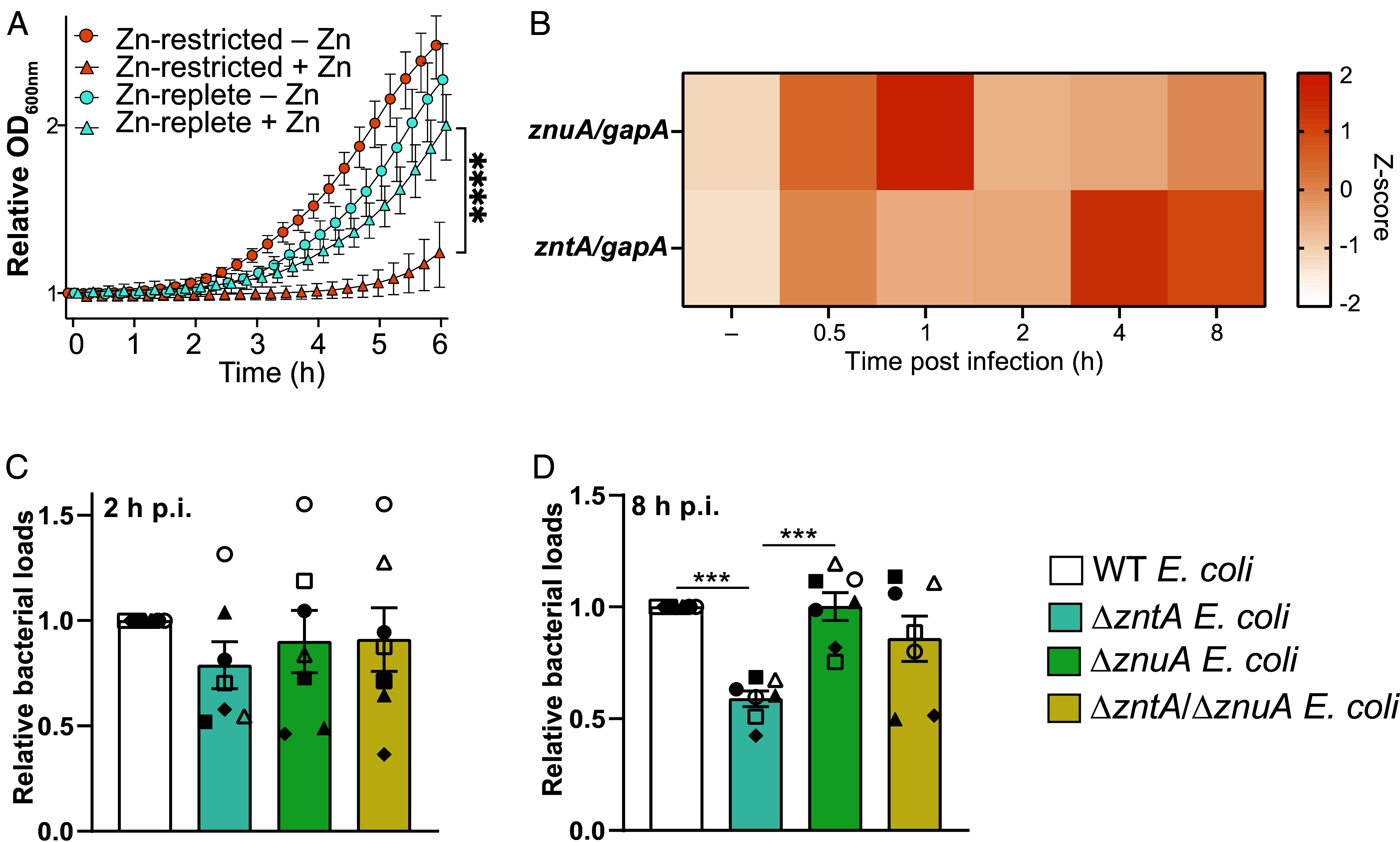
The zinc toxicity response against *E. coli* requires a functional zinc acquisition system. (*A*) WT *E. coli* was cultured in LPM medium containing 31.25 µM ZnSO_4_ (zinc replete), or no Zn (zinc starved). Bacteria were washed and resuspended in LPM media supplemented with 0 (−Zn) or 31.25 µM (+Zn) ZnSO4. Optical density was measured every 15 min for 6 h. Data represent mean ± SD of three (n = 3) independent experiments, plotted relative to T_0_ of each condition. Data were analyzed by two-way ANOVA with Tukey’s multiple comparisons test to compare effects of zinc treatment, **** denotes *P* < 0.0001. (*B*) HMDM were infected with WT *E. coli* for an 8 h time course, after which *znuA* or *zntA* mRNA levels were assessed by qPCR. Control samples (−) represent bacteria cultured in macrophage media for 0.5 h. Data are presented as heatmap of the mean of six (n = 6) independent experiments. (*Cand D*) HMDM were infected with WT, Δ*zntA*, Δ*znuA,* or Δ*zntA*/Δ*znuA E. coli* and intracellular bacterial loads were assessed at 2 h p.i. (*C*) and 8 h p.i. (*D*). Data are shown relative to WT *E. coli* at each time point. Data are presented as mean + SEM from seven (n = 7) independent experiments, with each experiment designated by a different symbol (square, circle, triangle, etc.). Data were analyzed by one-way ANOVA with Tukey’s multiple comparison test. *** denotes *P* < 0.001, all other comparisons were not significant.

**Fig. 6.**
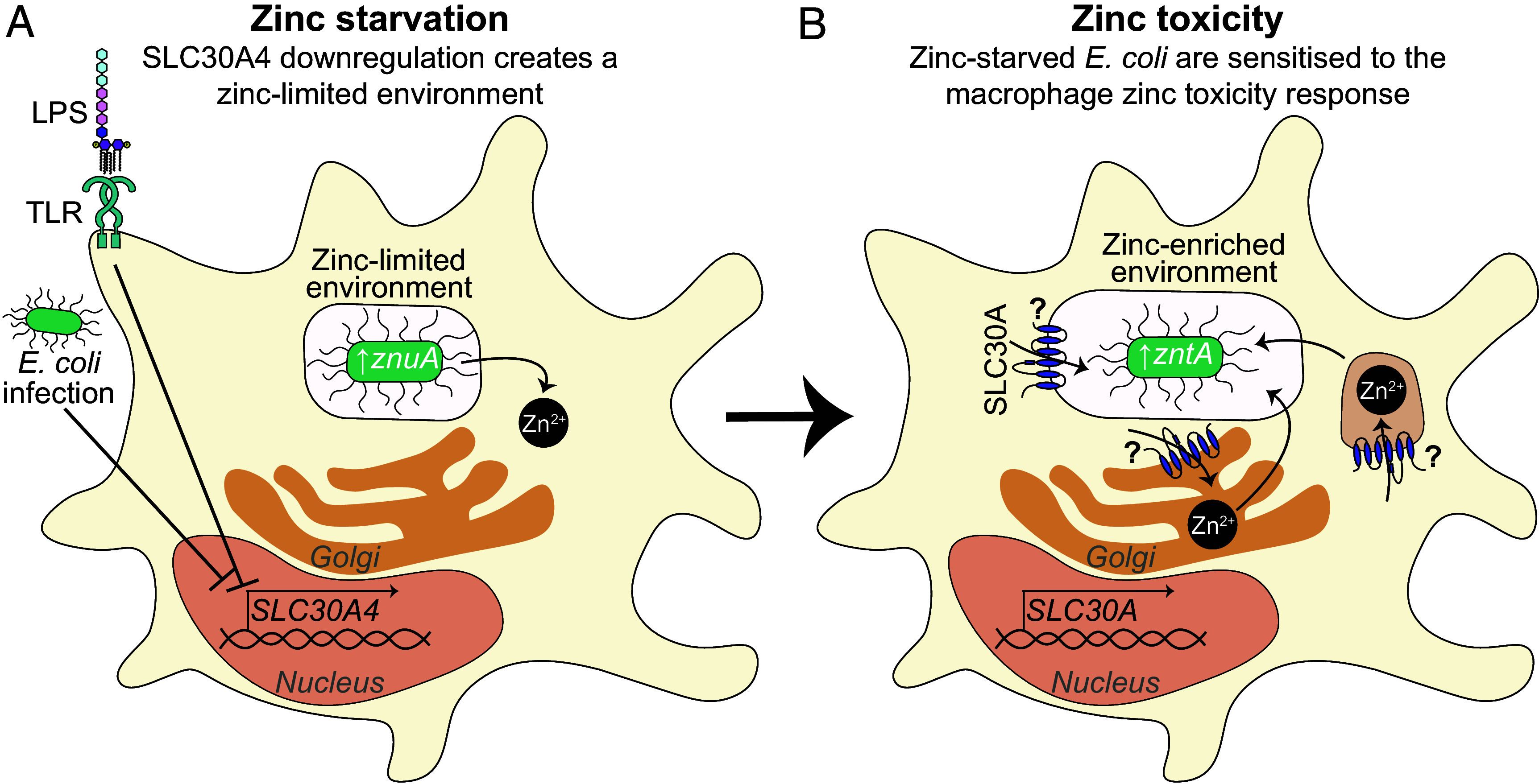
SLC30A4: a potential molecular switch for zinc-related antimicrobial responses. (*A*) LPS stimulation or *E. coli* infection downregulates *SLC30A4* mRNA expression in macrophages. This, and likely other mechanisms, starves pathogen-containing compartments of zinc, resulting in inducible *znuA* expression to permit bacterial survival. This adaptation by *E. coli* to a zinc-limited environment sensitizes them to the subsequent zinc toxicity response. (*B*) Macrophages mobilize zinc to phagosomes, for example, through SLC30A1 that localizes to intracellular zinc-containing vesicles (13). Zinc mobilization toward bacteria in the phagosome results in a zinc stress response against intracellular *E. coli*, which is countered by inducible *zntA* expression. Intracellular survival of *E. coli* is reduced in the absence of *zntA*, with this effect requiring a functional *znuA*.

If our model above is correct, we would expect that zinc starvation of *E. coli* should increase sensitivity to zinc intoxication. To assess this in vitro, *E. coli* were cultured in low-phosphate, low-magnesium medium (LPM) to model the phagosomal environment within macrophages (25), either in zinc-replete or zinc-starved conditions, after which they were subsequently exposed to a high zinc environment. These experiments confirmed that prior exposure to a zinc-limited environment greatly increased the sensitivity of *E. coli* to zinc stress (Fig. 5*A* and *SI Appendix*, Fig. S7*C*). For example, concentrations below 250 µM ZnSO_4_ only partially inhibited the growth of *E. coli* that had previously been cultured in the presence of 31.25 µM zinc, whereas all these ZnSO_4_ concentrations completely prevented the growth of *E. coli* that had previously been cultured under zinc-limited conditions (*SI Appendix*, Fig. S7*C*). Next, we assessed our proposed model that zinc starvation by macrophages sensitizes intracellular *E. coli* to subsequent zinc toxicity by examining zinc-linked gene signatures of *E. coli* within macrophages. Here, we found that *znuA* mRNA was rapidly induced in HMDM at 1 h p.i., whereas upregulation of *zntA* mRNA peaked later at 4 to 8 h p.i. (Fig. 5*B* and *SI Appendix*, Fig. S7*D*). This suggests that *E. coli* become sequentially subjected to zinc limitation and zinc intoxication in the intramacrophage environment. To determine whether zinc limitation was required for the zinc toxicity response against *E. coli* in HMDM, we assessed survival of a zinc-sensitive (Δ*zntA*) mutant, a zinc uptake mutant (Δ*znuA*), and a double mutant deficient in both zinc uptake and export (Δ*zntA*/Δ*znuA*). While similar bacterial loads were observed for all strains at 2 h p.i. (Fig. 5*C*), intracellular survival of the Δ*zntA* mutant was significantly reduced at 8 h p.i. (Fig. 5*D* and *SI Appendix*, Fig. S7*E*). However, *znuA* inactivation on the Δ*zntA* background attenuated the zinc susceptibility phenotype in macrophages from five of the seven donors that were examined (Fig. 5*E*). Collectively, these data support a model in which macrophages sequester zinc away from intracellular *E. coli* by downregulating SLC30A4 and/or other mechanisms, with this countered by *E. coli* through the deployment zinc acquisition systems (Fig. 6*A*). Consequently, this host-dependent reprogramming of intracellular *E. coli* makes them susceptible to subsequent zinc intoxication by macrophages (Fig. 6*B*).

## Discussion

In this study, we leveraged the heterogeneity of the HMDM zinc toxicity response (Figs. 1*D* and 2*C*) to identify potential regulators of this antibacterial pathway. Our HTBRP scRNA-seq approach identified host genes associated with macrophage zinc toxicity, including both known zinc regulators (*SLC30A4*, *MT1H,* and *MT1G*) and others not previously linked to zinc biology (e.g., *S100A10* and *TPT1*). Two subpopulations of HMDM containing high levels of zinc-stressed bacteria were enriched for zinc and metal ion pathways in their transcriptomes (Fig. 3*A*), thus validating our approach. Moreover, one of these clusters (cluster 10^6h^) was enriched for *SLC30A1*. This gene encodes SLC30A1/ZNT1 that was previously shown to surround intracellular *E. coli* in macrophages, and to promote zinc stress and bacterial killing (13). The gene expression program of the other cluster (cluster 2^6h^) that was enriched for *SLC30A4* may permit intracellular survival of zinc-stressed *E. coli*, given our findings from functional studies on SLC30A4. Future studies on genes selectively enriched in clusters 2^6h^ and 10^6h^ (Datasets S2 and S4) should reveal additional mechanisms by which macrophages engage and/or attenuate zinc-mediated antibacterial defense. For example, in addition to *SLC30A1*, several metallothionein-encoding genes (*MT1E*, *MT1F*, *MT1G*, *MT1H*, *MT1M*, *MT1X*, *MT2A*), *TMEM50B* that encodes an intracellular transmembrane protein (26), and the Rho family GTPase *RHOU* were all enriched in cluster 10^6h^ and thus represent candidate mediators of zinc toxicity for further investigation.

In focusing on cluster 2^6h^ and SLC30A4, our studies revealed an unexpected connection between genetic signatures associated with macrophage zinc toxicity and zinc limitation experienced by intracellular *E. coli* during infection, thus providing insights into potential interaction of these antimicrobial pathways in these key innate immune cells (Fig. 6). Previous studies have demonstrated differential engagement of innate immune zinc starvation and zinc toxicity responses in different contexts. In a murine infection model, *Acinetobacter baumannii* was subjected to zinc starvation in the respiratory tract and zinc intoxication in the spleen, revealing differences in the zinc response to infection in different tissues (27). Similarly, neutrophils deployed zinc starvation as an antimicrobial response against extracellular *Streptococcus pyogenes* but zinc toxicity upon phagocytosis (28). Here, we provide evidence for cross talk between these seemingly opposing antimicrobial pathways within the context of intracellular host defense. *E. coli* zinc starvation and zinc toxicity signatures were both induced in HMDM at the population level (Fig. 5*B*) and the susceptibility of a zinc-sensitive mutant to clearance by macrophages was dependent on the ZnuA zinc acquisition system (Fig. 5*D*). Consistent with these findings, *E. coli* grown in medium approximating the phagosomal environment (25) and under zinc limitation were dramatically sensitized to zinc intoxication (Fig. 5*A* and *SI Appendix*, Fig. S7*C*). These observations raise the question of whether bacterial pathogens that resist and/or evade macrophage zinc toxicity, for example, *Salmonella* (9) and uropathogenic *E. coli* (10), might achieve such defense by manipulating the zinc starvation response.

We hypothesized that genes identified through our screen may either contribute to the zinc toxicity pathway or facilitate survival of zinc-stressed bacteria. Subsequent functional studies on SLC30A4 suggest that this gene belongs to the latter class (Fig. 4). The known roles of SLC30A4 in macrophage antimicrobial responses depend on context. SLC30A4 colocalized with phagosomes and supported survival of the fungal pathogen *Histoplasma capsulatum* in IL-4-stimulated mouse macrophages (22), whereas it and SLC30A7/ZNT7 were implicated in zinc sequestration in the Golgi for effective host defense against *H. capsulatum* in granulocyte macrophage-colony stimulating factor-stimulated murine macrophages (23). We found that overexpression of SLC30A4 increased “available” zinc, as assessed by Fluozin-3 staining (Fig. 4*C* and *SI Appendix*, Fig. S6*B*). This likely reflects SLC30A4-mediated delivery of zinc to the Golgi (*SI Appendix*, Fig. S6*C*) (23) and/or phagosomes (22), rather than increased uptake of extracellular zinc (*SI Appendix*, Fig. S6*D*). Given that SLC30A4 overexpression impaired zinc starvation of *E. coli* (Fig. 4*G*), the dramatic downregulation of *SLC30A4* in HMDM responding to *E. coli* (Fig. 4*F*) likely contributes to the zinc starvation response. However, given the acute upregulation of *znuA* mRNA in *E. coli* within macrophages that we observed (Fig. 5*B*), other more direct mechanisms are also likely involved. Whatever the precise mechanisms, our data are consistent with sensitization of zinc-starved bacteria to zinc intoxication by macrophages (Figs. 5 and 6). We conclude that the enrichment of *SLC30A4* mRNA expression in HMDM harboring zinc-stressed bacteria (cluster 2^6h^: Figs. 2*C* and 3 *C* and *D*) represents a failure of this population to effectively engage zinc starvation. This would be expected to facilitate survival of intracellular *E. coli* that had subsequently been exposed to zinc stress. Additional functional studies on *SLC30A4* and other genes enriched in cluster 2^6h^ should reveal additional insight into how engagement of zinc starvation sensitizes intracellular bacteria to the macrophage zinc toxicity response.

Transcriptional profiling of bacterial gene expression programs within mammalian cells has been informative for understanding intracellular environmental niches, including in relation to metal ion availability (29). Previous studies have also used paired sequencing to interrogate host pathogen interactions, either through bulk RNA-seq (30–32) or low-throughput single-cell RNA-seq (33, 34). Recently, a method utilizing fluorescent reporter *Salmonella* Typhimurium containing scRNA-seq barcodes on mRNA PolyA tails was used, alongside fluorescent sorting of host cells, to study host–pathogen interactions in murine bone marrow–derived macrophages (35). Our approach complements this study, through the adaptation of 10× Genomics feature barcode technology to capture bacterial responses within a high-throughput, mammalian-optimized RNA sequencing platform. This enabled us to track the human macrophage zinc toxicity response against *E. coli* at a single-cell level in the absence of pathogen evasion. This approach should also be amenable to quantifying intracellular pathogen gene expression profiles at the whole transcriptome level, thus enabling interrogation of multiple antimicrobial pathways and/or host subversion strategies. Our reporter-based workflow could also be applied to single-cell proteomics that has similarly been used to investigate macrophage heterogeneity (36).

The current study has several limitations. First, in accordance with ethics requirements, we had no knowledge of age, sex, and other factors that may confound data interpretation for the human donors used in this study. The heterogeneity in the zinc toxicity response that was apparent between HMDM from different donors (Fig. 1*D*) suggests that there is likely to be variability in different macrophage populations with respect to interplay between zinc starvation and zinc intoxication. This is supported by our bacterial infection assays where *znuA* deficiency on the *zntA* background compromised antibacterial defense in HMDM from five out of seven donors (Fig. 5*D*). Second, the mechanisms used by macrophages to engage zinc starvation are likely to extend beyond *SLC30A4* downregulation. Currently, we have a limited understanding of these mechanisms. Third, our scRNAseq approach will not capture all genes/proteins that promote or inhibit the macrophage zinc toxicity response, such as lowly expressed genes and/or proteins that may be posttranslationally modified to engage the pathway. Indeed, our analysis did not identify *CFTR* that is required for zinc intoxication by human macrophages (15). Thus, mediators of this antibacterial pathway will undoubtedly extend beyond the candidate genes that we identified in this study (Datasets S2 and S4). Finally, we used a nonpathogenic *E. coli* K-12 strain to study antibacterial defense in macrophages in the absence of pathogen evasion strategies. Future studies should address whether bacterial pathogens that target macrophages, such as *Salmonella* and *Mycobacteria*, subvert zinc starvation to overcome macrophage-mediated zinc toxicity.

The global burden of antibiotic resistance (37) necessitates alternative approaches to combat infections caused by bacterial pathogens, for example, host-directed therapies (38). An understanding of molecular mechanisms that either drive innate immune antibacterial responses or facilitate intracellular bacterial survival can help guide such approaches. For example, our studies suggest that targeting of SLC30A4 may enhance host defense against bacterial pathogens. More broadly, strategies aimed at augmenting zinc starvation of bacteria by macrophages might be expected to enhance the effectiveness of the zinc toxicity response. In summary, our use of HTBRP scRNA-seq has uncovered both heterogeneity and complexity in molecular mechanisms driving the macrophage zinc toxicity response. The tools and methods generated in this study should have broad utility for understanding host–pathogen dynamics and in shaping strategies for anti-infective design.

## Materials and Methods

### *E. coli* Strains, Culturing, and Zinc Starvation Assays.

The *E. coli* K-12 strain MG1655 (39) and the uropathogenic *E. coli* strain EC958 (40) were used in this study. Modification of our previously described zinc dual reporter plasmid pGcCzntAp (10) was performed by Epoch Life Science (USA). All recombinant strains and plasmids are described in *SI Appendix*, Table S1. Mutants were made using the λ-Red recombinase system, as previously described (40). Bacteria were routinely cultured at 37 °C in liquid or solid Lysogeny Broth (LB) medium containing relevant antibiotics for plasmid maintenance (30 mg/mL chloramphenicol, 100 mg/mL ampicillin). To assess the effect of zinc starvation on *E. coli* susceptibility to zinc in conditions approximating the intramacrophage environment, *E. coli* were cultured in LPM medium (8 µM MgCl_2_, 5 mM KCl, 7.5 mM NH_4_SO_4_, 0.5 mM K_2_SO_4_, 0.3% v/v glycerol, 0.1% casamino acid, 337 μM H_2_PO_4_, 80 mM 2-(N-morpholino) ethanesulfonic acid, pH 5.8) medium (25) supplemented with or without 31.25 µM ZnSO_4_. Bacteria were then washed and recultured in LPM media containing 0 to 2 mM ZnSO_4_, with optical density (600 nm) measured every 15 min for 6 h.

### HMDM Culturing.

All work involving primary human cells was approved by The University of Queensland Medical Research Ethics Committee (Human Research Ethics Approval numbers: 2013001519, 2022/HE002118). Anonymized human buffy coats were provided by Australian Red Cross Lifeblood, with donors providing informed consent. Monocytes were isolated and differentiated into HMDM for 6 to 7 d with 150 ng/mL recombinant human colony-stimulating factor (rhCSF-1, produced in-house, Protein Expression Facility, UQ), as previously described (10). Adherent HMDM were harvested before seeding onto TC-treated plates for subsequent experimentation as described. In some experiments, cells were stimulated with 100 ng/mL LPS from *S. enterica* serotype Minnesota L2137 (Sigma-Aldrich), as indicated in individual figures.

### Gene Overexpression in THP-1 Cells.

Gene overexpression in macrophages was achieved via a Dox-inducible lentiviral system, as previously described (41, 42), utilizing pF_TRE3G_PGK_puro (pLenti_EV) kindly provided by James Murphy (Walter and Elizabeth Hall Institute of Medical Research, Melbourne). pLenti_EV was modified by Gene Universal (Newark, DE) to include V5-tagged genes of interest within the MCS. Details on methods used for lentiviral-based gene overexpression and expression constructs can be found in *SI Appendix*, Table S2 and *Supplementary Methods*.

### Infection Assays.

Bacteria were cultured in antibiotic-free LB broth at 37 °C overnight. Infection of lentiviral-transduced THP-1 cells was performed after antibiotic removal as described above and HMDM were plated in antibiotic-free media. Macrophages were infected at indicated multiplicity of infection (MOI) for 1 h. HMDM used for HTBRP scRNA sequencing were spin-infected (MOI 30,500 g for 5 min at 37 °C), before incubation 37 °C for 55 min. Gentamicin exclusion of extracellular bacteria was maintained for the remainder of infection assays. Intracellular bacterial loads were quantified by lysing macrophages with 0.1% Triton X-100 (Sigma-Aldrich) in PBS, followed by incubating on LB agar overnight at 37 °C. Colonies were counted and CFU/mL was calculated.

### Confocal Microscopy and Quantification.

HMDM were seeded onto 12 mm coverslips (Thermo Fisher Scientific) and then infected. Coverslips were fixed with 4% paraformaldehyde (Sigma-Aldrich) in PBS, then stained with 20 ng/mL DAPI, mounted onto slides, and imaged at 63× 1.4 NA plan apochromat objective using a Zeiss LSM710 Meta fitted to an Axio Examiner upright microscope (Carl Zeiss, Jena, Germany), running Zeiss Zen Black 2012 software. To quantify intracellular bacteria, a custom written, user-interactive ImageJ macro “Bugfinder script” was codeveloped and written by Nicholas Condon (IMB, UQ, https://github.com/NickCondon/BugFinderAssign). Briefly, images were split into DAPI, GFP, and mCherry single channels, with the following performed: background subtraction (rolling ball, radius = 10), median filtering (radius = 2), and thresholding (DAPI = Huang, Bacteria = Max Entropy) were automatically applied. Objects greater than 30 µm^2^ from the DAPI channel were identified as “macrophage nuclei.” GFP objects were segmented using local maxima (prominence = 50), and those greater than 0.15 µm^2^ were identified as “bacteria.” Measurements were recorded for all identified objects, including the maximum, median, and mean fluorescent intensities of GFP and mCherry channels and exported. Bacteria were classified as “mCherry^Positive^” or “mCherry^Negative^” based on threshold mCherry intensities measured from intramacrophage MG1655-GFP *E. coli* single-color controls in each experiment. DAPI^Positive^/GFP^Negative^/mCherry^Negative^ bacteria were considered as nonviable and excluded from analysis. The shortest hypotenuse between each nuclei and bacteria’s center of mass GFP coordinate was used to assign bacteria to individual macrophage nuclei, which was confirmed visually in region of interest overlays. For confocal microscopy on transfected THP-1 cells, 1 × 10^5^ cells were plated over coverslips in a 24-well plate, fixed in 4% paraformaldehyde for 15 min, then washed three times with PBS. For immunofluorescence, cells were incubated with blocking buffer (5% FCS, 0.3% Triton X-100 in PBS) for 1 h. The blocking buffer was then replaced with the anti-V5 primary antibody (Bio-Rad) diluted in the blocking buffer and incubated for 1 h. After washing with PBS, cells were incubated in anti-mouse Alexa Fluor 647 secondary antibody (Thermo Fisher Scientific) and DAPI for 1 h. For Fluozin-3 staining, cells were incubated with Fluozin-3 (5 µM) for 30 min, followed by DAPI incubation for 20 min. Cells were washed three times with PBS, and the coverslips were mounted using IMBiol mounting media (Gelvatol mounting media, IMB). Subsequently, slides were imaged using a Zeiss Axiovert 200 Inverted Microscope (Zeiss).

### ICP-MS.

ICP-MS was used to quantify total levels of zinc and other metal ions within transfected THP-1 cells. In brief, 10 × 10^6^ PMA-differentiated THP-1 cells transfected with vehicle or *SLC30A4* mRNA were washed at 16 h posttransfection with 5 mL Hanks’ Balanced Salt Solution (HBSS; without Mg^2+^ and Ca^2+^, Gibco). Cells were then lysed in 5 mL of freshly prepared 0.1% (w/v) SDS in H_2_O, then transferred to preweighed ICP-MS–compatible tubes (provided by School of the Environment, The University of Queensland). After transferring the lysates, tubes were reweighed and 200 µL of triple-distilled HNO_3_ (School of the Environment, The University of Queensland) was added to acidify the sample to a final concentration of 2%. Tubes were weighed again, and samples were adjusted to a total volume of 10 mL with Milli-Q water and final tube weights were recorded. Prepared samples were analyzed for biometals using an Agilent 7900 Inductively-Coupled Plasma Mass-Spectrometer (Agilent). No cell (media only) and lysis buffer controls were included to account for background, and the levels of biometals (Zn, Mg, Mn, Fe, and Cu) were calculated following background subtraction.

### Flow Cytometry.

RPMI containing zinc sulfate heptahydrate (Sigma-Aldrich) at indicated concentrations was inoculated with overnight bacterial cultures and incubated as described. At indicated time points, infected HMDM were enzymatically lifted from 6-well TC plates in TrypLE Express (Thermo Fisher Scientific). Differentiated and Dox-induced THP-1 cells were lifted in lift buffer [PBS + 0.1% sodium azide (Sigma-Aldrich), 2 mM EDTA] and fixed in 1% formaldehyde for 1 to 4 h and washed thrice in PBS (centrifugation at 1,000 g for 3 min). To stain intracellular labile zinc, THP-1 cells were stained in 1:1,000 FluoZin-3 (Thermo Fisher Scientific) in PBS. To assess overexpression of V5-tagged SLC30A4, fixed THP-1 cells were permeabilized (PERM buffer [1% FCS, 0.25% Saponin (Sigma-Aldrich), 5 mM EDTA in PBS]) and blocked in Trustain FcX (Biolegend), before incubation with 1 µg/mL mouse anti-V5 primary antibody (Bio-Rad) and then 2 µg/mL chicken anti-mouse Alexa 647 (Invitrogen) secondary antibody. All cells were washed and resuspended in PBS before assessment on an CytoFLEX S (Beckman Coulter) or LSR Fortessa flow cytometer (Becton Dickinson Pty. Ltd., BD), using optic and filter configurations indicated in *SI Appendix*, Table S3. Debris and doublets were excluded. Single color *E. coli* strains MG1655-GFP, pGc_EV MG1655, and MG1655-mCherry (*SI Appendix*, Table S1) were used as compensation controls. Data were analyzed using CytExpert (Beckman Coulter) or FlowJo (BD) software.

### HTBRP scRNA-seq.

HMDM were spin-infected with barcoded zntR-dual reporter *E. coli* (*SI Appendix*, Table S1). HMDM were enzymatically lifted in TrypLE Express and washed. HMDM viability was assessed to be >80% by Trypan blue exclusion counted on a Countess^TM^ 3 FL Automated Cell Counter (ThermoFisher). Standard reagents (excluding the oligo-dT reverse transcription primer) were prepared for a modified 5’ scRNA-seq 10× Genomics workflow (*SI Appendix*, Fig. S2*C*, Chromium Single Cell 5’ Reagent Kits User Guide CG000331 [v2 Chemistry Dual Index]), with additional reagents included for the indicated purpose: 333 U ReadyTM Lyse Lysozyme and 0.32 mM Ambion® EDTA, to aid bacterial lysis; 40 μM random hexamer (RH) primer with PCR handle (Integrated DNA Technologies, Iowa) to allow reverse transcription of mammalian and bacterial transcripts; and 30 U Vaccinia capping enzyme (NEB), 1.3× capping buffer (NEB), 0.8 mM GTP (NEB), 0.8 mM SAM (NEB), and 0.004 U yeast pyrophosphatase (NEB), to encourage template switching during reverse transcription of bacterial transcripts. Microdroplets were achieved by passing HMDM through the 10× Genomics Chromium controller and an additional reaction of 42 °C for 60 min was performed to modify bacterial RNA transcripts with 5’ caps. Following reverse transcription, a 10× cell barcode and UMIs were incorporated into full-length cDNA through the template switching oligo (TSO). Feature barcode *GFP* and *mCherry* transcripts were reverse transcribed via TSO targeting capture sequences engineered into the reporter plasmid barcode sequence, thereby allowing incorporation of a 10× cell barcode and UMI. Recovery agent and Dynabeads cleanup was performed. cDNA was minimally amplified using primers mix targeting full-length and feature barcode cDNA, and fractions were separated by size selection. ZapR rRNA depletion (Takara Bio Inc., Shiga, Japan) was performed on full-length cDNA. Full-length and feature barcode cDNA outputs were amplified, then used for gene expression and *GFP*/*mCherry* feature barcode library preparation, respectively. Libraries were pooled and sequenced via an Illumina NovaSeq SP 100 cycle kit using the following run configurations: Read1 26 bp, Index I 10 bp, Index 2 10 bp, Read2 90 bp.

### Analysis of scRNA-seq Data.

Briefly, raw RNA-sequencing reads were processed by CellRanger v4.00 and v6.0.1 (10× Genomics) and aligned to GRCh38 (GENCODE v32/Ensemble 98) reference to generate output barcode and feature matrices. Data were mainly analyzed using multiple packages in R v4.0.4 (R Foundation for Statistical Computing, Vienna, Austria). Empty droplets were filtered based on the feature counts using DropletUtils v1.10.3. Next, doublets were removed using scDblFinder v1.4.0. Outliers were excluded based on the number of genes detected, quantity of transcripts, and percentage of mitochondrial transcripts using Seurat v4.0.4 (43). Filtered feature counts were normalized via SCTransform in Seurat v4.0.4 with regularized negative binomial regression to remove technical noise (44). Dimension reduction was performed by principal component analysis and the top 30 principal components were selected for clustering and UMAP two-dimensional projections via DimPlot function in Seurat. The distributions of genes of interest were plotted using FeaturePlot function provided by Seurat. Macrophage labeling was performed using Label Transfer tutorial in Seurat, based on reference datasets previously defined for hematopoietic cell types (19). Violin plots were generated through Seurat function VlnPlot. *mCherry* and *GFP* correlation was performed in R v4.0.4 using Pearson correlation in function “cor.” RNA velocity analysis (21) was performed using velocyto v0.17 and scVelo v0.2.4 on Python v3.8.8 (Python Software Foundation, Oregon) for Seurat clusters. Three-dimensional (3D) projections were generated using plotly v4.10.0 on Python. DEG lists were generated using Seurat function Findmarkers. Biological pathways were identified through assessment of DEGs via GO Resource’s GO Enrichment Analysis (http://geneontology.org/) (45–47).

### Statistical Analyses.

Statistical analyses were performed using GraphPad Prism 7 software (Graphpad, San Diego). For experiments involving HMDM, each experiment used cells isolated from a different donor. Data from single or duplicate experiments are represented as indicated, and statistical analyses were not performed. *P* values of 0.05 or less were statistically significant.

### Detailed Methodologies.

Details of all bacterial strains, plasmids, primers, antibodies, and flow cytometer configurations, along with detailed methodologies for gene overexpression in THP-1 cells, western blotting, and qPCR are provided in *SI Appendix*.

## Supplementary Material

Appendix 01 (PDF)

Dataset S01 (XLSX)

Dataset S02 (XLSX)

Dataset S03 (XLSX)

Dataset S04 (XLSX)

## Data Availability

The full single-cell RNA datasets are available via NCBI’s Sequence Read Archive (BioProject accession: PRJNA1121940) (48). Details for barcoded reporter plasmids are available upon request. All other data are included in the manuscript and/or supporting information.
