# Supplementary material for: Bacterial reporter–paired scRNA sequencing reveals cross talk between zinc starvation and zinc toxicity in macrophage antibacterial defense
Source: Proc Natl Acad Sci U S A. 2026 Mar 9;123(11):e2530503123. doi: 10.1073/pnas.2530503123 (PMC12993976; doi:10.1073/pnas.2530503123)
Supplement: Supplementary file 1 — Appendix 01 (PDF) [file pnas.2530503123.sapp.pdf]

PNAS

Supplementary Information for

Bacterial reporter-paired scRNA sequencing reveals crosstalk between zinc starvation and zinc toxicity in macrophage antibacterial defense.

Jessica B. von Pein[1,2], Stacey B. Andersen[3,4], Jon Xu[3,4], Minh-Duy Phan[1,5,6], Emma K. Dalton[1,2,6], Michael Koczerka[7], Claudia J. Stocks[1,2,6], James E. B. Curson[1,2,6], Zoe Vandeleur [1,2,6], Nicholas D. Condon[1], Steven J. Hancock[1,5,6], Christian M. Nefzger[1,2], Nathan J. Palpant[1,2], Divya Ramnath[1,2,6], Ronan Kapetanovic[1,2,7,*], Mark A. Schembri[1,5,6,*] and Matthew J. Sweet[1,2,6,*].

[1]Institute for Molecular Bioscience (IMB), The University of Queensland (UQ), Brisbane, Queensland Australia 4072; [2]IMB Centre for Cell Biology of Chronic Disease, UQ, Brisbane, Queensland Australia 4072; [3]Genome Innovation Hub, UQ, Brisbane, Queensland Australia 4072; [4]UQ Sequencing Facility, UQ, Brisbane, Queensland Australia 4072; [5]School of Chemistry and Molecular Biosciences, UQ, Brisbane, Queensland Australia 4072; [6]Australian Infectious Diseases Research Centre, UQ, Brisbane, Queensland Australia 4072; [7]INRAE, Université de Tours, ISP 1282, Nouzilly, France, 37380; [*]Co-corresponding authors..

*Co-corresponding authors: Matthew J. Sweet, Mark A. Schembri, Ronan Kapetanovic

Email: m.sweet@imb.uq.edu.au, m.schembri@uq.edu.au, ronan.kapetanovic@inrae.fr

This PDF file includes:

Supporting text – Supplementary Methods
SI Figures 1-7
SI Tables 1-5
SI References

Supplementary Methods

Gene overexpression in THP-1 cells

Lentiviral transduction was performed to introduce pLenti MCE (Empty Vector, EV) or pLenti_SLC30A4_V5 into THP-1 cells, as described previously (1, 2). Briefly, Lipofectamine 2000 (Invitrogen) in OptiMEM (Gibco) was combined with packaging plasmid (pCMV-dR8.2 dvpr [Addgene; Cambridge, USA]), an envelope plasmid (pCMV_VSV-G [Addgene]) and the relevant DNA transfer plasmid (pLenti_EV or pLenti_SLC30A4_V5). Transfection mixtures were added to media of Human embryonic kidney (HEK) 293T cells (3), and viral supernatants were collected after 24 hours and applied to THP-1 cells (obtained from the ATCC) 1,000 *g* spinfection for 100 min at 35 ºC. THP-1 cells were then placed under 1 µg/mL puromycin (Sigma-Aldrich) to select for lentivirally-transduced cells. After expansion, lentiviral THP-1 cells were seeded for experimentation and differentiated for 48 h with 30 ng/mL phorbol-12-myristate-13-acetate (PMA, Sigma-Aldrich). Adherent THP-1 cells were recovered in PMA-free culturing media in the final ~3 h of differentiation (4). 100 ng/mL of doxycycline for 16 h, followed by a 4 h antibiotic-free recovery, was confirmed to induce overexpression of SLC30A4 in lentiviral THP-1 cells. Subsequent experiments proceeded as described. As an alternative approach for gene overexpression, THP-1 cells were transfected with an mRNA encoding SLC30A4. Human *SLC30A4* mRNA sequence was acquired from NCBI. Codon optimization was performed using mRNAid (5), and the sequences were inserted between incomplete 5' and 3' UTRs designed for expression by the BASE Facility (The University of Queensland). DNA templates (synthesized by GenScript) were used in PCR reactions, from which mRNAs with complete UTRs were *in vitro* transcribed using the HiScribe™_T7 mRNA kit with CleanCap reagent AG (New England Bioscience) as per the manufacturer's instructions, except standard UTP was substituted with 5-methoxy UTP (Thermo Fisher Scientific Milwaukee) to reduce immunogenicity (6). mRNA was purified using a Monarch RNA clean up kit (NEB) as per the manufacturer's instructions. PMA-differentiated THP-1 cells were allowed to recover for 3 h in PMA-free media before transfection with mRNA (1 µg/million cells) using Lipofectamine messenger max (Thermo Fisher Scientific) as per the manufacturer's protocol, then seeded for experimentation as indicated.

Western Blotting

Cells were lysed in RIPA buffer (50 mM Tris-Cl, 150 mM NaCl, 0.1% SDS (Bio-Rad), 1% sodium deoxycholate, 1% NP-40, pH 7.4), containing 1x phosphatase inhibitor Cocktail (Sigma-Aldrich) and 1x protease inhibitor cocktail (Roche Diagnostics, Indianapolis, USA). Cell lysates were homogenised, then prepared with 1x reducing agent (Invitrogen) and 1x lithium dodecyl sample buffer (Life Technologies). Samples were electrophoresed on 4-12% Mini Protean SDS-PAGE precast gels (Bio-Rad) and transferred onto methanol-activated Immobilon-P PVDF membranes (Millipore). Membranes were blocked, incubated in primary and secondary antibodies (SI Table 4), then visualized using Clarity ECL (Bio-Rad) on a ChemiDoc™ MP (Bio-Rad). Membranes were inactivated with 30% H_2O_2.

qPCR

Cells were lysed in RLT buffer (Qiagen), total RNA was extracted using RNeasy® Mini Kit (Qiagen) and 1 µg of mRNA was reverse transcribed using Superscript III (Invitrogen). For mammalian and bacterial gene expression, on-column DNase I treatment (Qiagen) and Oligo dT (Invitrogen), or Turbo DNA-free (Invitrogen) and random hexamers (Thermo Fisher Scientific) were applied, respectively. mRNA was quantified via qPCR on Applied Biosystems 7900HT fast qPCR system using primers (SI Table 5) purchased from Sigma-Aldrich, Integrated DNA Technologies and/or Geneworks (Hindmarsh, Australia). Normalisation was performed by the ΔC_t method (7) relative to hypoxanthine phosphoribosyl transferase (*hHPRT*, mammalian) or *gapA* (bacterial).

SI Figures and Tables

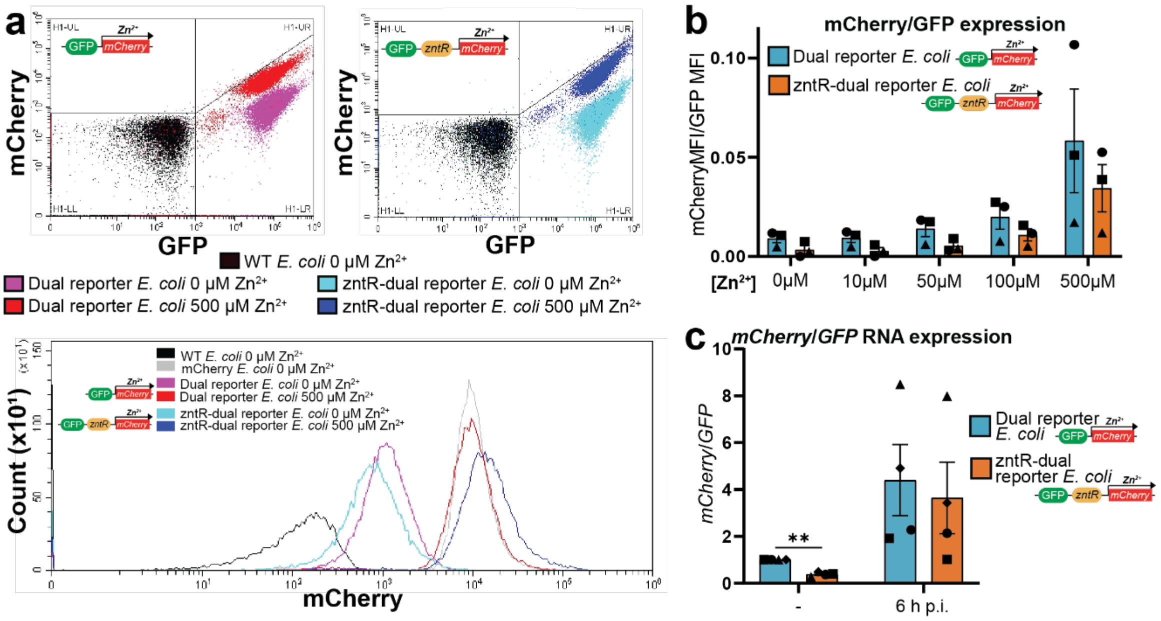

SI Figure 1: zntR-dual reporter *E. coli* has reduced basal *mCherry* expression than dual reporter *E. coli*. (a-b) WT, dual reporter and zntR-dual reporter *E. coli* were cultured in the absence or presence of zinc (Zn^{2+}) for 6 h and analysed by flow cytometry. **(a)** Dot plots and histograms of mCherry signal of bacterial populations from a single representative experiment of three (n=3) independent biological replicates. Quadrant gates were set based on mCherry signal from dual reporter *E. coli* - Zn^{2+}. **(b)** mCherry median fluorescence intensity (MFI) normalised to GFP MFI are shown combined from three (n=3) independent biological replicates. Statistical analysis was performed by two-way ANOVA with Bonferroni multiple comparisons test, all comparisons were not significant. **(c)** HMDM were infected with WT, dual reporter and zntR-dual reporter *E. coli* for 6 h. Control samples (-) represent bacteria cultured in complete RPMI 1640 media for 2 h. *mCherry* and *GFP* RNA expression was quantified by qPCR from five (n=5) independent experiments. **(b, c)** Graphed data represent mean ± SEM, with individual experiments represented with different symbols (circle, square, triangle and diamond). Two-way ANOVA with Bonferroni multiple comparisons test was applied. **=p<0.01, all other comparisons not significant.

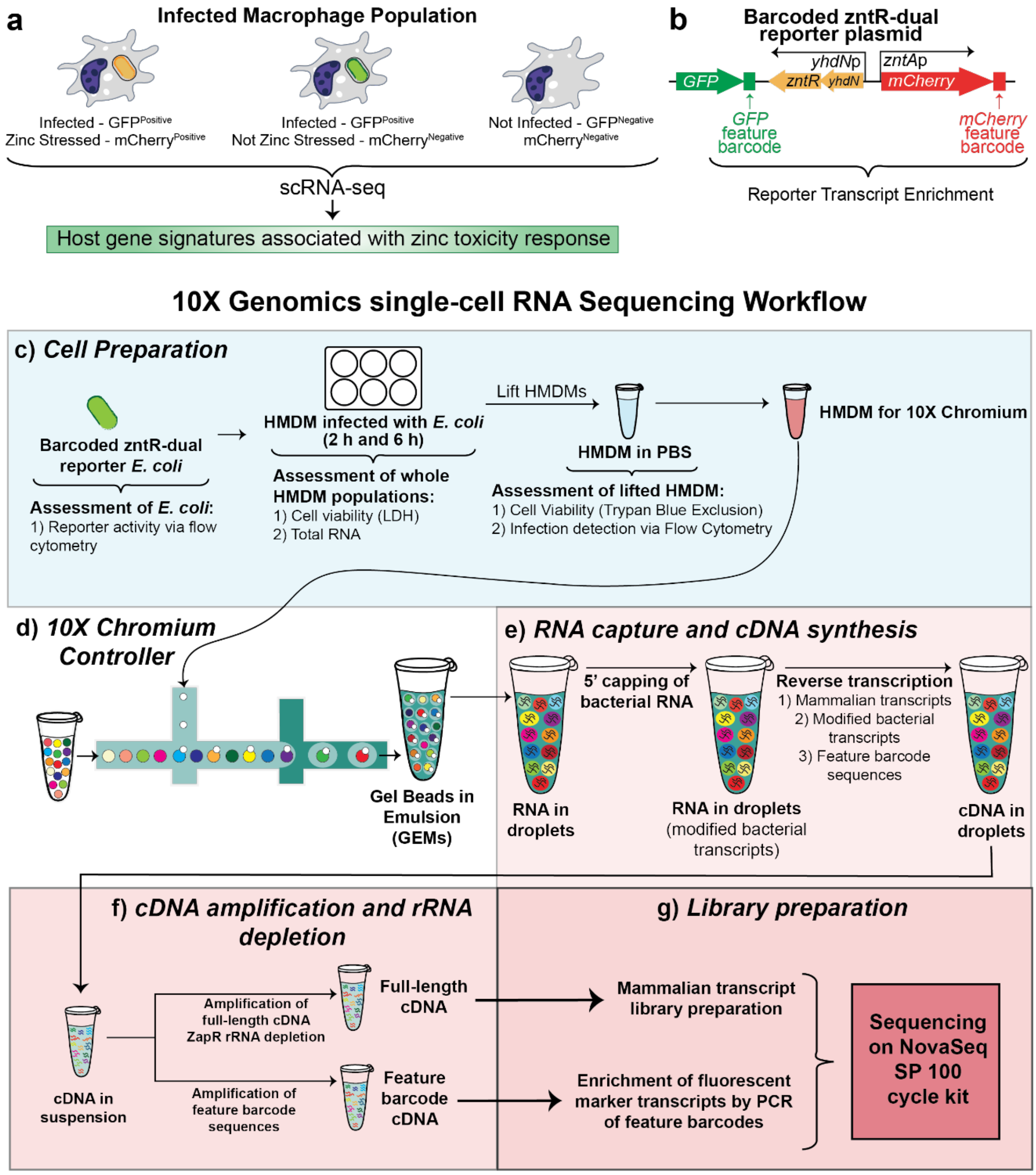

SI Figure 2: A high-throughput bacterial-paired single-cell RNA sequencing method to capture native host and bacterial zinc stress reporter transcripts. (a) Reporter *E. coli* *GFP* and *mCherry* profiles within HMDM during infection. **(b)** Feature barcodes in the barcoded zntR-dual reporter construct allows for specific amplification of *GFP* and *mCherry* transcripts during single-cell RNA sequencing. **(c)** HMDM were spin-infected with MOI 30 barcoded zntR-dual reporter *E. coli* for 2 or 6 h. Cell viability and total population RNA were assessed from matched infected and uninfected HMDM populations. Lifted HMDM were washed and added to 10x Genomics 5' v2 reaction mix containing additional lysozyme, EDTA and capping reagents, before HTBRP scRNA-seq via the 10x Genomics Chromium Controller **(d)**. **(e)** 5' capping of bacterial RNA and reverse transcription reactions were performed. (f) Full-length cDNA and feature barcode cDNA were amplified separately. **(g)** Gene expression libraries from full-length cDNA and feature barcode libraries were prepared from feature barcode cDNA, pooled and sequenced with an Illumina NovaSeq SP 100 cycle kit.

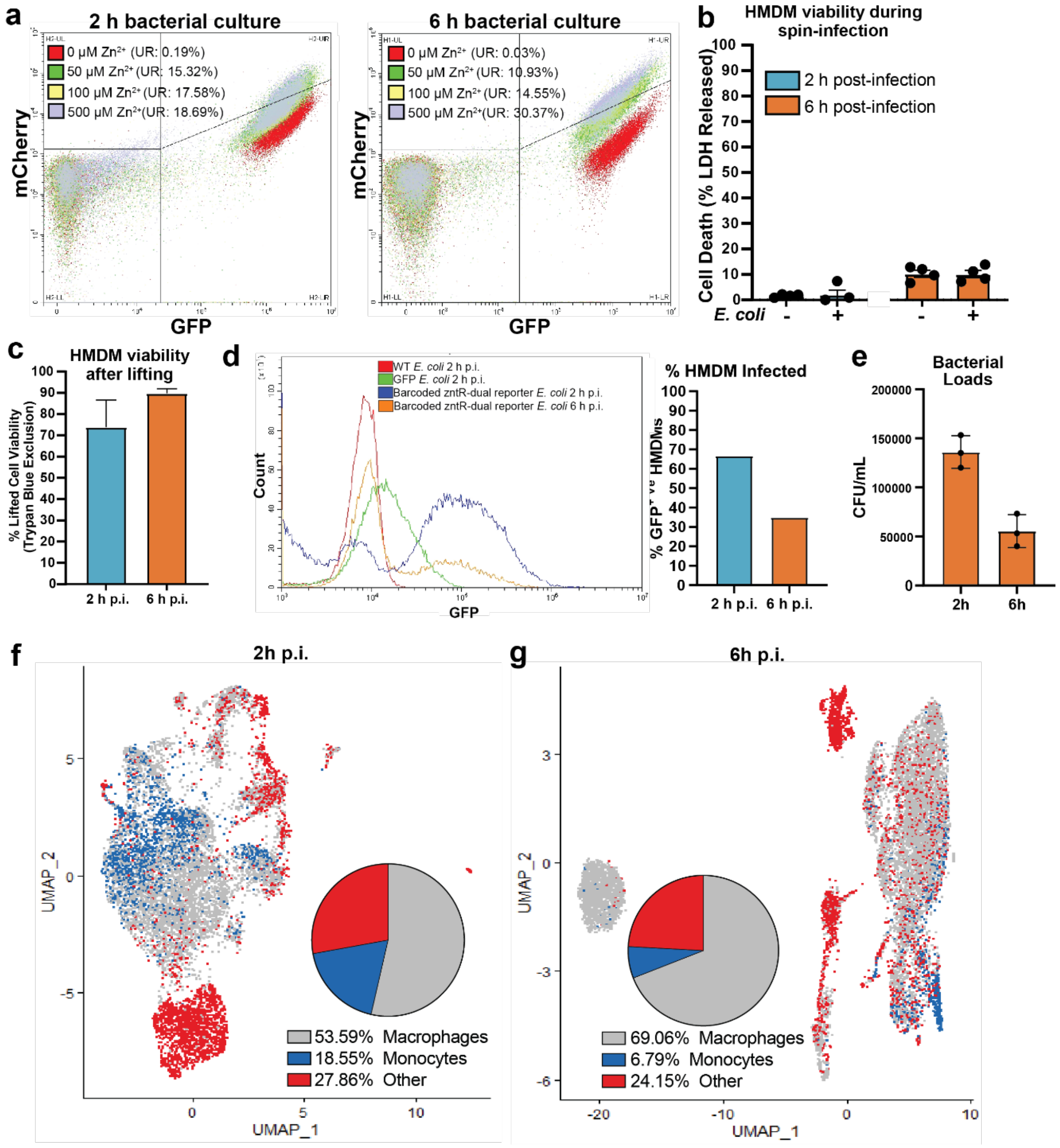

SI Figure 3: Validation of HMDM populations for interrogation via HTBRP scRNA-seq. **(a)** Barcoded zntR-dual reporter *E. coli* cultures used for HTBRP scRNA-seq were cultured in RPMI containing Zn^{2+} then assessed via flow cytometery. Dot plots of bacterial populations are taken from a single experimental replicate (n=1). The percentage of bacteria located in the Upper Right (UR) quadrant are indicated for each treatment. **(b-d)** HMDM populations matched with populations infected for HTBRP scRNA-seq were assessed from a single experiment (n=1). **(b)** HMDM viability was assessed via LDH release assay. Data show mean % LDH released ± SD from four technical replicates per condition. **(c)** Viability of lifted infected HMDM was assessed via Trypan Blue exclusion on a haemocytometer. Data shown represent mean % of live cells ± SD from four and nine technical replicates for 2 h and 6 h conditions, respectively. **(d)** Lifted infected HMDM used for HTBRP scRNA-seq were assessed via flow cytometry for bacterial GFP intensity. A histogram of GFP signal intensity and the % of cells with $GFP^{Positive}$ (GFP^{+ve}) signal are presented. **(e)** Lifted HMDM which were used for HTBRP scRNA-seq (at 2 and 6 h post-infection) were lysed and intracellular *E. coli* viability was assessed by plating on LB agar, and counting colony forming units (CFU). **(f-g)** Gene expression libraries obtained from HMDM infected with barcoded zntR-dual reporter *E. coli* for 2 h (n = 9,864 HMDM) and 6 h (n = 9,496 HMDM) were sequenced and data were

processed with a human reference. HMDM within each dataset were labelled according to marker genes previously defined for haematopoietic cell types (8) and applied to UMAP projections.

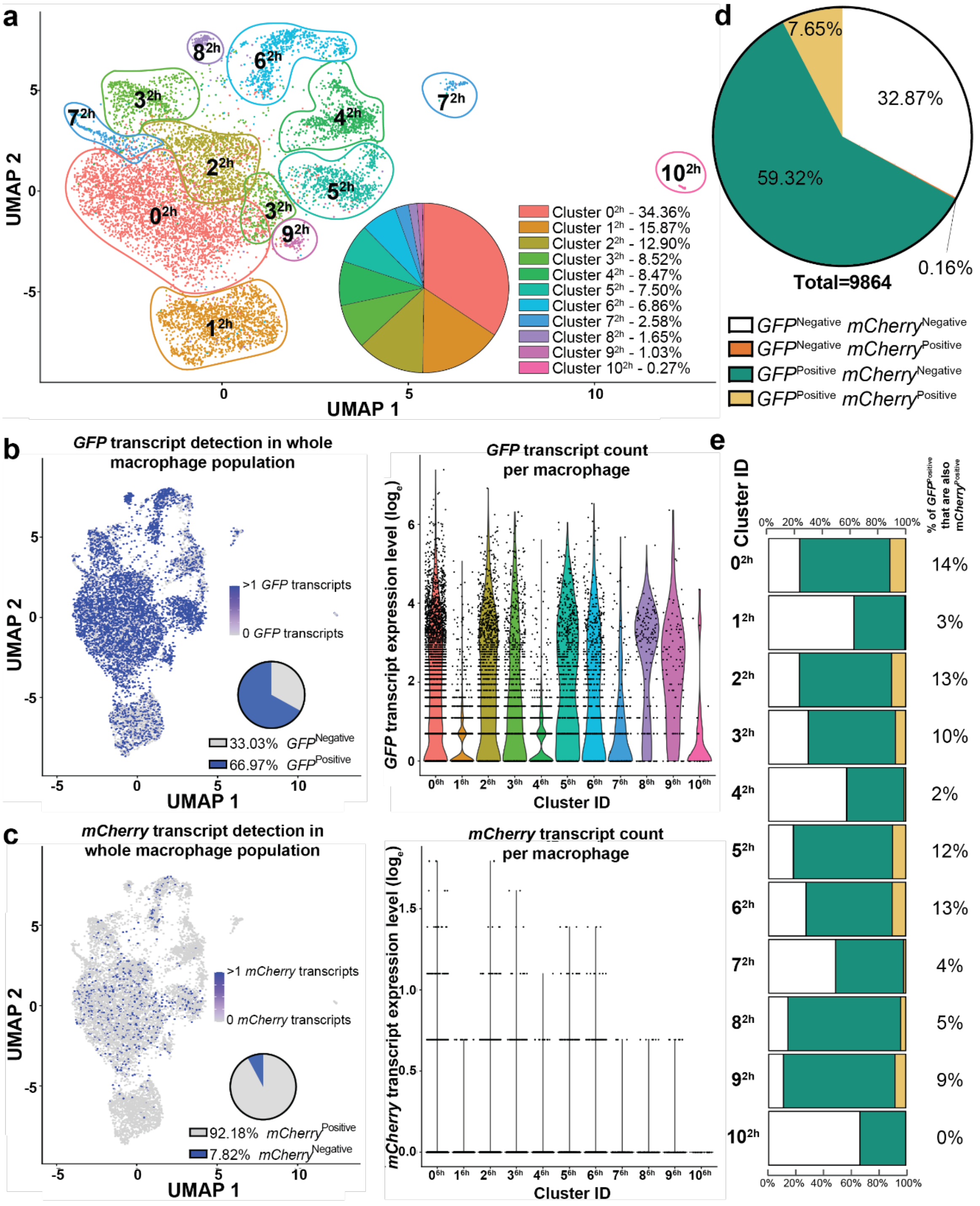

SI Figure 4: HTBRP scRNA-seq to track zinc toxicity at 2 h post-infection. HTBRP scRNA-seq and analysis was performed on gene expression libraries from HMDM infected with barcoded zntR-dual reporter *E. coli* (MOI 30) for 2 h. **(a)** Two-dimensional UMAP of human gene expression and proportions of the overall HMDM population represented in each UMAP cluster. The distribution of HMDM *GFP* **(b, left)**, and *mCherry* **(c, left)** transcripts were overlayed onto gene expression data with % of $GFP^{Positive}$ $mCherry^{Positive}$ HMDM in the total population shown within pie-chart insets. Variation of *GFP* **(b, right)** and *mCherry* **(c, right)** transcript counts (log transformed) from individual HMDM, grouped according to UMAP clusters as defined in **(a)**. **(d-e)** Bacterial GFP and mCherry expression profiles detected within single HMDM are represented as percentages of macrophages in the total population

(d), and within UMAP clusters in bar schematics **(e)**. The percentage of *GFP*^{Positive} HMDM that are *mCherry*^{Positive} are indicated on the right of each cluster in **(e)**.

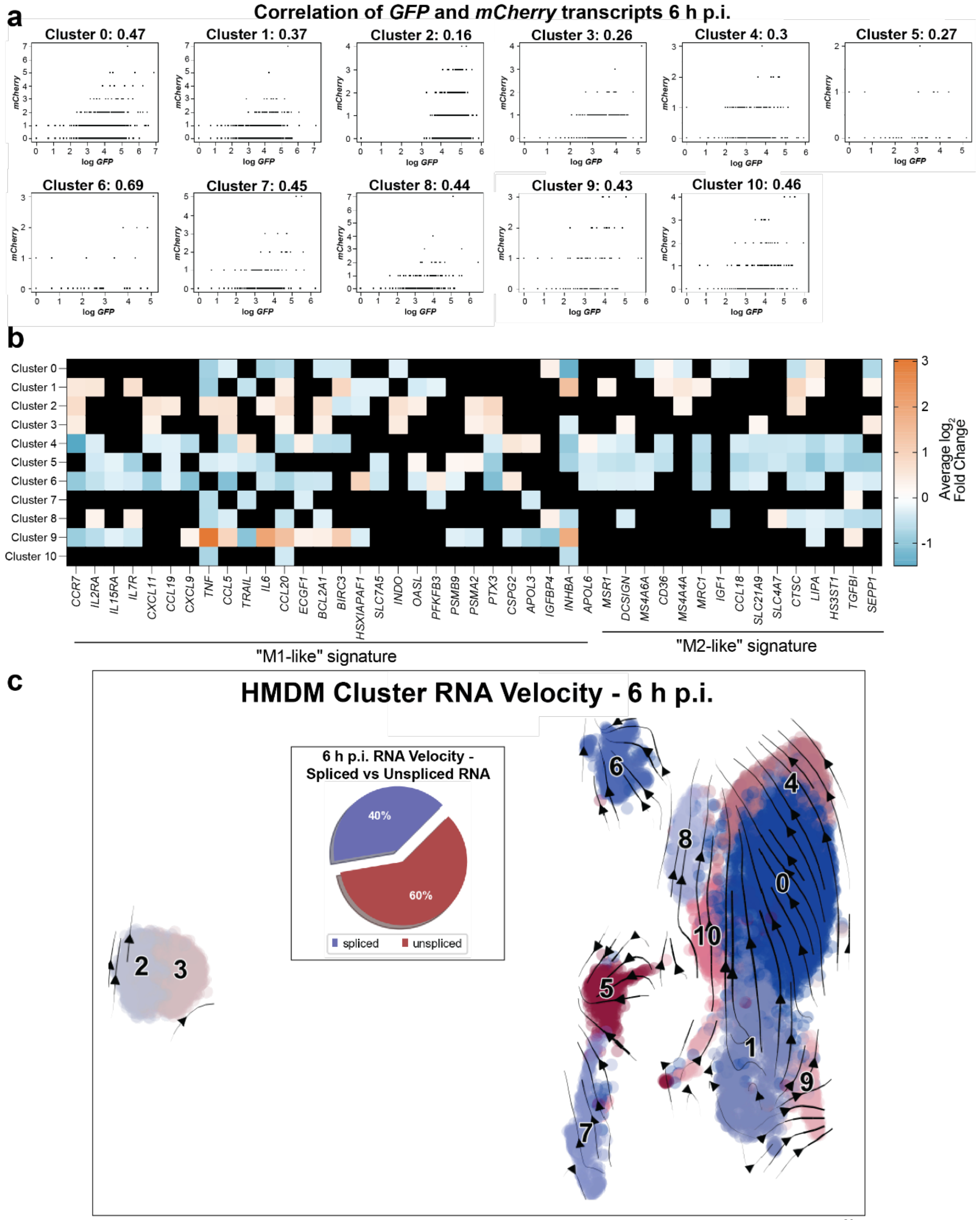

SI Figure 5: *mCherry* and GFP transcript counts do not correlate, and cluster 2^{6h} and cluster 3^{6h} represent distinct HMDM states. Pooled gene expression, and *GFP* and *mCherry* feature barcode libraries obtained from HMDM infected with barcoded zntR-dual reporter *E. coli* for 6 h were sequenced and data were processed. **(a)** Correlation analysis of *GFP* (log transformed) and *mCherry* transcript counts for individual HMDM within UMAP clusters (**Fig 2a**) are presented in individual plots. Correlation coefficients for each cluster are indicated above each plot. **(b)** Heatmap of DEG profiles within infected HMDM clusters, according to pro-inflammatory and anti-inflammatory signatures that have previously been defined (9). Genes not identified as a DEG in clusters/comparisons are represented with a

black square. **(c)** Velocity field contours determined by RNA velocity analysis are presented over UMAP clusters. The inset shows the proportion of spliced and unspliced RNA.

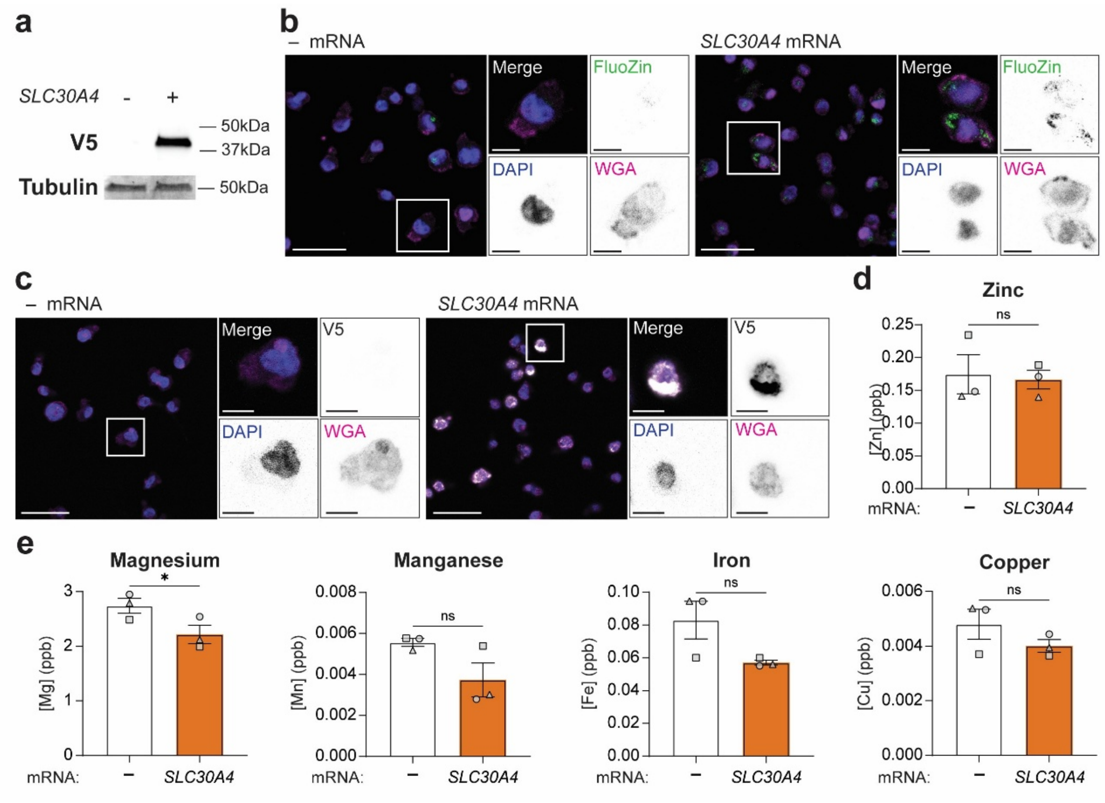

SI Figure 6: Overexpression of SLC30A4 causes a redistribution of intracellular zinc but does not alter total intracellular zinc concentrations. PMA-differentiated THP-1 cells were transfected with *SLC30A4* mRNA or vehicle control (-) for 16 h. **(a)** Immunoblot of THP-1 lysate, representative of three experiments (n=3). **(b-c)** Transfected THP-1 cells were fixed and stained for Fluozin-3 **(b)** or V5 **(c)** and assessed via confocal fluorescence microscopy. Images shown are representative of three independent experiments (n=3). Scale bars = 40 µm or 10 µm (inset). **(d-e)** Intracellular zinc and other metal ion concentrations in transfected THP-1 cells were analyzed by ICP-MS. Data represent mean ± SEM metal ion concentration normalized to BCA analysis for three (n=3) independent experiments. Data was analyzed by two-tailed T-test *=<0.05, ns=not significant.

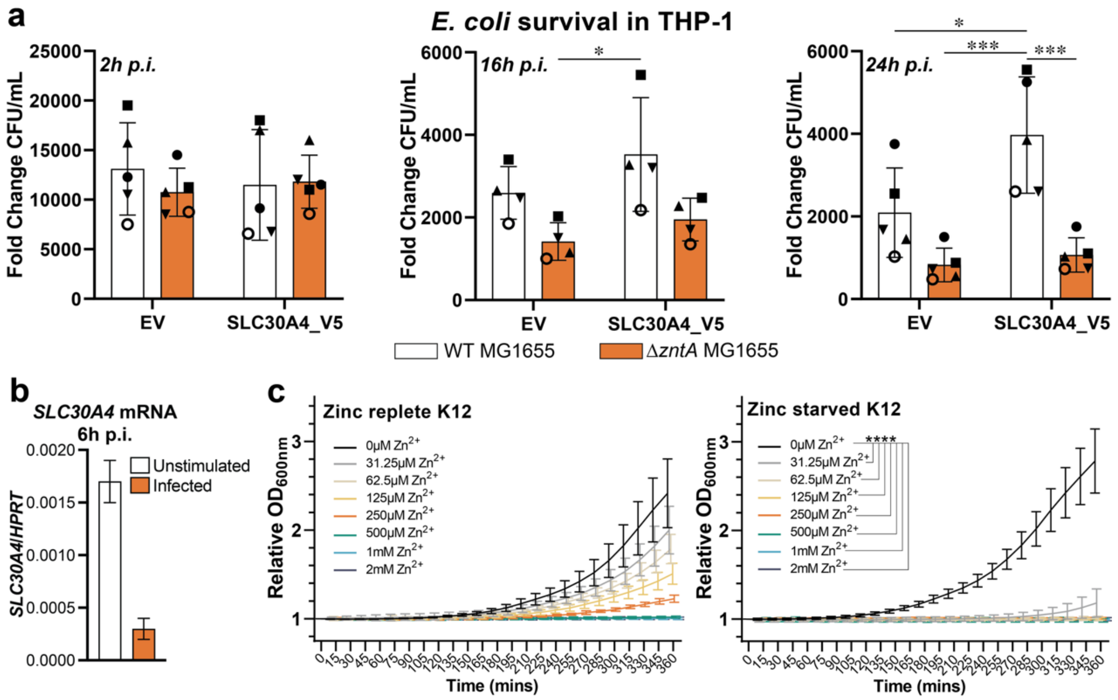

d

Time p.i. (h)	*znuA/gapA* mean±SEM (fold change)	*zntA/gapA* mean±SEM (fold change)
-	0.005±0.004 (1)	0.0013±0.0006 (1)
0.5	0.098±0.07 (19)	0.104±0.073 (75)
1	0.17±0.14 (33)	0.064±0.017 (46)
2	0.036±0.018 (7)	0.069±0.03 (50)
4	0.044±0.02 (8)	0.22±0.12 (159)
8	0.067±0.03 (13)	0.18±0.09 (133)

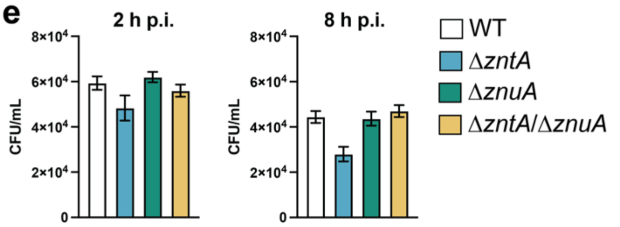

SI Figure 7: DEGs from HMDM subpopulations associated with the zinc toxicity response indicate functional gene candidates. (a) Doxycline-stimulated PMA-differentiated THP-1 cells (either empty vector (EV) or SLC30A4_V5) were infected with WT or Δ*zntA E. coli* and intracellular bacterial loads (CFU/mL) were assessed at indicated time points. Data represent mean ± SD of four to five (n=4-5) independent experiments, with each experiment indicated by different symbols. Two-way ANOVA with Bonferroni multiple comparisons test was performed. *=p<0.05 and ***=p<0.001, all other comparisons were not significant. **(b)** *SLC30A4*/*ZNT4* mRNA expression from total uninfected and infected HMDM populations matched with the population used for HTBRP scRNA-seq was determined by qPCR (relative to *HPRT*). Data represent mean ± SD from three technical replicates from a single HMDM donor (n=1). (**c**) WT K12 were cultured in LPM medium containing 31.25 μM $ZnSO_4$ (zinc replete) or no Zn (zinc starved). Bacteria were washed and resuspended in LPM media supplemented with 0-2 mM ZnSO4. Optical density was measured every 15 minutes for 6 h. Data represent mean ± SD of three (n=3) independent experiments, plotted relative to T_0 of each condition. Data were analysed by two-way ANOVA with Tukey's multiple comparisons test to compare effects of zinc treatment, **** denotes p<0.0001. (**d**) HMDM were infected with WT *E. coli*, after which *znuA* or *zntA* mRNA levels were assessed by qPCR. Control samples (-) represent bacteria cultured in macrophage media for 0.5 h. The table shows the mean ± SEM of six (n=6) independent experiments, with the fold change over bacteria alone in brackets. (**e**) HMDM were infected with WT, Δ*zntA*, Δ*znuA* or Δ*zntA*/Δ*znuA* *E. coli* and intracellular bacterial loads were assessed. Data represent mean + range of technical replicates from a single representative experiment (n=7).

SI Table 1. *E. coli* strains and plasmid descriptions.

Strain Reference Name	Strain/Plasmid Description	Source
**Wild type (WT) strains**		
MG1655	*E. coli* K-12 strain MG1655. Non-pathogenic.	(10)
EC958	Uropathogenic *E. coli* ST131 isolate	(11, 12)
**Fluorescent reporter strains**		
MG1655-GFP	Constitutive chromosomal *GFP* expression. Chloramphenicol resistance.	(13)
pGc_EV MG1655	Single-colour plasmid; Constitutive *GFP* expression driven by the *S.* Typhimurium *rpsM* promoter (p*rpsM*), and *mCherry* with no promoter driving expression. Chloramphenicol resistance.	
MG1655-mCherry	Single-colour plasmid; pFPV with constitutive *mCherry* expression; Ampicillin resistance.	Gift from Prof Kate Schroder (IMB, UQ).
Dual reporter MG1655	Dual-reporter plasmid; Constitutive *GFP* expression driven by p*rpsM*, and *mCherry* driven by p*zntA*. Chloramphenicol resistance. Derived from pFCcGi vector (14)	(13, 15)
zntR-dual reporter MG1655	ZntR-dual reporter plasmid; Constitutive *GFP* expression driven by p*rpsM*, and *mCherry* downstream of p*zntA*. *zntR* operon from CFT073 upstream of, and in reverse orientation to, p*zntA*. Chloramphenicol resistance.	This study.
Barcoded zntR-dual reporter MG1655	Barcoded dual-reporter plasmid for 5' scRNA-seq reactions; Constitutive *GFP* (C-terminal barcode sequence, see SI Fig 2b) expression driven by p*rpsM*, and *mCherry* (C-terminal barcode sequence) downstream of p*zntA*. *zntR* operon inserted upstream of, and in reverse orientation to, p*zntA*. Chloramphenicol resistance.	
**Zinc-sensitive strains**		
Δ*zntA* MG1655	Single mutant MG1655 achieved by λ red recombinase gene inactivation of *zntA*. Chloramphenicol resistance. Zinc-sensitive phenotype.	(13, 15, 16)
Δ*znuA* MG1655	Single mutant MG1655 achieved by λ red recombinase gene inactivation of *zntA*. Chloramphenicol resistance.	This study.
Δ*zntA*/ Δ*znuA* MG1655	Double mutant MG1655 achieved by λ red recombinase gene inactivation of *pstB*. Chloramphenicol resistance.	

SI Table 2. Primers used for confirmation of lentiviral constructs via sequencing.

Primer	Sequence	Purpose	Size (bp)
MCS Fwd	CGTATAAGCTTTAGGCGTGT	Confirmation of full insert sequences	MCS = 914
Lenti seq Rev	CTAGGGGAGGAGTAGAAGGT		
Lenti Seq Fwd	GAAGGTGGAGAGAGAGACAGAG		
SLC30A4_V5 Rev	CCTGAAGTGAGAGACCAGATATTTA		1332
SLC30A4_V5 Fwd	GGTGAAAGCCAGGTTGACC		

SI Table 3. Flow cytometer optic and filter configurations.

Flow Cytometer	Fluorophore/Dye	Laser	Filter
CytoFLEX S (Beckman Coulter)	mCherry	Yellow/Green (561 nm)	610/20
	GFP	Blue (488 nm)	525/40
	Alexa Fluor 647	Red (638 nm)	660/10
LSR Fortessa (BD)	FluoZin-3	Blue (488 nm)	530/30

SI Table 4. Antibodies used in western blot and immunofluorescence (IF).

Specificity	Description	Dilution (Concentration), Diluent	Source
Primary Antibodies			
GAPDH	Rabbit monoclonal anti-GAPDH	1:2500 (0.1 mg/mL), 5% skim milk in TBST – western blot	Cell Signalling Technology, Boston, USA
V5	Mouse anti-V5 tag	1:1000 (1 mg/mL), 5% skim milk in TBST – western blot<br>1:500 (0.5 mg/mL), 5% FCS, 0.3% Triton X-100 in PBS - IF	Bio-Rad
Secondary Antibodies			
Rabbit IgG HRP	Goat anti-rabbit IgG HRP	1:3000 (0.48 mg/mL), 5% skim milk in TBST – western blot	Cell Signalling Technology
Mouse IgG HRP	Horse anti-mouse IgG HRP	1:3000 (0.48 mg/mL), 5% skim milk in TBST – western blot	
Mouse Alexa 647	Donkey anti-Mouse IgG Alexa Fluor™ Plus 647	1:400 in 5% FCS, 0.3% Triton X-100 in PBS - IF	Thermo Fisher Scientific

SI Table 5. Primers used for qPCR in this study

Gene Target	Primer Type	Sequence	Amplicon Size (bp)
gapA (*E. coli*)	Fwd	GGTGCGAAGAAAGTGGTTATGAC	88
	Rev	GGCCAGCATATTTGTCGAAGTTAG	
zntA (*E. coli*)	Fwd	CGAAGCACAGGTTGCTGAAC	107
	Rev	CGCAAATCAATACGCGCTCA	
znuA (*E. coli*)	Fwd	GGCCAACCCAAACGACTAAG	140
	Rev	GTTCATCGCTTCTGCCATTG	
mCherry	Fwd	AAGTAGTCGGGGATGTCGGC	130
	Rev	GCACCCAGACCGCCAAG	
GFP	Fwd	TGGAGAGGGTGAAGGTGATGC	122
	Rev	AGCATTGAACACCATAACCGAAAGT	
hHPRT	Fwd	TCAGGCAGTATAATCCAAAGATGGT	84
	Rev	AGTCTGGCTTATATCCAACACTTCG	
hSLC30A4	Fwd	AAGCGCCATCATACTCACCC	99
	Rev	GCTGACAAAACCTCTAAGCGA	

SI References

1. J. M. Murphy *et al.*, The pseudokinase MLKL mediates necroptosis via a molecular switch mechanism. *Immunity* **39**, 443-453 (2013).
2. J. E. Vince *et al.*, IAP antagonists target cIAP1 to induce TNFalpha-dependent apoptosis. *Cell* **131**, 682-693 (2007).
3. R. B. DuBridge *et al.*, Analysis of mutation in human cells by using an Epstein-Barr virus shuttle system. *Mol Cell Biol* **7**, 379-387 (1987).
4. M. E. Lund, J. To, B. A. O'Brien, S. Donnelly, The choice of phorbol 12-myristate 13-acetate differentiation protocol influences the response of THP-1 macrophages to a pro-inflammatory stimulus. *J Immunol Methods* **430**, 64-70 (2016).
5. N. Vostrosablin *et al.*, mRNAid, an open-source platform for therapeutic mRNA design and optimization strategies. *NAR Genom Bioinform* **6**, lqae028 (2024).
6. H. Moradian, T. Roch, L. Anthofer, A. Lendlein, M. Gossen, Chemical modification of uridine modulates mRNA-mediated proinflammatory and antiviral response in primary human macrophages. *Mol Ther Nucleic Acids* **27**, 854-869 (2022).
7. M. W. Pfaffl, A new mathematical model for relative quantification in real-time RT-PCR. *Nucleic Acids Res* **29**, e45 (2001).
8. Z. Bian *et al.*, Deciphering human macrophage development at single-cell resolution. *Nature* **582**, 571-576 (2020).
9. F. O. Martinez, S. Gordon, M. Locati, A. Mantovani, Transcriptional profiling of the human monocyte-to-macrophage differentiation and polarization: new molecules and patterns of gene expression. *J Immunol* **177**, 7303-7311 (2006).
10. F. R. Blattner *et al.*, The complete genome sequence of Escherichia coli K-12. *Science* **277**, 1453-1462 (1997).
11. M. Totsika *et al.*, Insights into a multidrug resistant Escherichia coli pathogen of the globally disseminated ST131 lineage: genome analysis and virulence mechanisms. *PloS one* **6**, e26578-e26578 (2011).
12. B. M. Forde *et al.*, The complete genome sequence of Escherichia coli EC958: a high quality reference sequence for the globally disseminated multidrug resistant E. coli O25b:H4-ST131 clone. *PLoS One* **9**, e104400 (2014).
13. C. J. Stocks *et al.*, Uropathogenic *Escherichia coli* employs both evasion and resistance to subvert innate immune-mediated zinc toxicity for dissemination. *Proceedings of the National Academy of Sciences* **116**, 6341-6350 (2019).
14. R. Figueira, K. G. Watson, D. W. Holden, S. Helaine, Identification of *Salmonella* pathogenicity island-2 type III secretion system effectors involved in intramacrophage replication of *S. enterica* serovar typhimurium: implications for rational vaccine design. *mBio* **4**, e00065-00013 (2013).
15. C. J. Stocks *et al.*, Frontline Science: LPS-inducible SLC30A1 drives human macrophage-mediated zinc toxicity against intracellular *Escherichia coli*. *Journal of Leukocyte Biology* **109**, 287-297 (2021).
16. R. Kapetanovic *et al.*, Salmonella employs multiple mechanisms to subvert the TLR-inducible zinc-mediated antimicrobial response of human macrophages. *The FASEB Journal* **30**, 1901-1912 (2016).
